# The spatial spillover effect of environmental regulation on the total factor productivity of pharmaceutical manufacturing industry in China

**DOI:** 10.1038/s41598-022-15614-8

**Published:** 2022-07-08

**Authors:** Qijie Wu, Yuexin Li, Yufei Wu, Fei Li, Shen Zhong

**Affiliations:** 1 School of Marxism Studies, Harbin University of Commerce, Harbin, Heilongjiang People’s Republic of China; 2 School of Finance, Harbin University of Commerce, No.1, Xuehai Street, Songbei District, Harbin, Heilongjiang People’s Republic of China; 3 School of Economics, Beijing Technology and Business University, Beijing, People’s Republic of China

**Keywords:** Environmental social sciences, Health care

## Abstract

As an important embodiment of a country's economic strength and national health, pharmaceutical manufacturing industry has made rapid development in China in recent years. But at the same time, the pharmaceutical manufacturing industry is facing many environmental problems, such as large pollution emissions, complex pollution components, controlling difficulties and so on. This paper measures the total factor productivity of pharmaceutical manufacturing industry (HTFP) by using data envelopment analysis with unexpected output, which is more accurate and effective than the traditional model. It also studies the effect of environmental regulation on the total factor productivity of pharmaceutical manufacturing industry (HTFP) by establishing panel data regression model and spatial econometric model based on 30 provinces in China from 2004 to 2019, which enriches the research results in the field of cleaning in pharmaceutical manufacturing industry. The conclusions are as follows: (1) Environmental regulation and total factor productivity of pharmaceutical manufacturing industry have significant spatial autocorrelation, showing "high-high" or "low-low" spatial aggregation characteristics; (2) Environmental regulation has a significant promoting effect on improving pharmaceutical manufacturing total factor productivity in local and surrounding areas, and there are differences in the impact of eastern, central and western regions; (3) Green technology, production technology and industrial structure play an important role in the impact of environmental regulation on pharmaceutical manufacturing total factor productivity, which provides theoretical guidance and policy recommendations for improving the level of total factor productivity of pharmaceutical manufacturing industry in the environmental aspect.

## Introduction

Pharmaceutical manufacturing industry is one of the high-tech industries, which only reflects the national economic strength, but also closely related to the health level of the people^1,2^. In early 2020, the COVID-19 swept across the world, which once again proved the importance of the development of pharmaceutical manufacturing industry^3^. In recent years, China's pharmaceutical manufacturing industry has made rapid development, with the total profit of China's pharmaceutical manufacturing industry reaching 369.3 billion yuan in 2020, an increase of 341.8 billion yuan compared with 2004. It has achieved nearly 13 times growth (*Statistical Yearbook of China's High-tech Industry*, 2005–2021). However, it cannot be ignored that the pharmaceutical manufacturing industry is facing serious pollution problems while giving full play to the advantages of knowledge-intensive and advanced technology. The pollution problems such as large pollution emissions, complex pollution components, are difficult to degrade and harmful to organisms, which are serious for environment^4^. Therefore, environmental regulation is very important to effectively alleviate the pollution problem of pharmaceutical manufacturing industry, which is promoting the sustainable development of pharmaceutical manufacturing industry.

China has a vast territory, and the degree of environmental regulation among provinces is not a big gap, as follows: First, the government is the main body of the implementation of environmental regulation policy. The government should adopt different environmental regulation policies according to the geographical location, economic development and environmental governance of each province^5^. There are some differences in the degree of government investment in environmental protection and environmental importance among provinces^6^. Second, manufacturing enterprises are one of the objects of environmental regulation policy. There are some gaps in the degree of industrialization of enterprises in different provinces, and there are some differences in the weight of the three industries. Moreover, due to the infrastructure construction, economic development and the degree of scientific and technological transformation in different provinces, there are also some differences in the speed and scale of green transformation of manufacturing enterprises among provinces. This will further increase the differences in environmental regulation among provinces^7,8^. Third, the degree of environmental regulation will be affected by the public environment. In a broad sense, the public environment includes the natural environment and the human environment. The degree of pollution of the natural environment can directly affect the degree of environmental regulation. In the humanistic environment, information disclosure and public participation have a certain degree of impact on environmental regulation policy^9^. Because of the differences of geographical location, natural conditions, cultural education, religious beliefs and so on, it will lead to the differences of public environment among provinces, which results in the differences of environmental regulation.

In addition, from the perspective of the impact of environmental regulation on the total elements of pharmaceutical manufacturing industry, the impact mechanism of spatial effect cannot be ignored^10^. Pharmaceutical manufacturing enterprises in provinces are affected by environmental regulation, which will increase production costs. On the one hand, the enterprises can choose to reduce scale and factor investment. So that labor and other factors of production flow outward. In this case, the first flow is to the adjacent areas, resulting in changes in the total factor productivity of the local and adjacent areas^11^. On the other hand, the enterprise can choose to carry out technological innovation, intensify research and development of clean technology, improve production technology, and adopt clean technology and equipment for production. Promote the technological upgrading of enterprises in the region. Technology has a certain spillover effect. The first benefit is the adjacent areas, thus affecting the total factor productivity of local and surrounding areas^12^.

Due to the different stages of environmental regulation policies and the development of pharmaceutical manufacturing industry, there are some differences in the impact of environmental regulation in different countries. This paper provides a way of thinking for the study of different countries in the world, it is very important to study the spatial effect of environmental regulation on the total factor productivity of pharmaceutical manufacturing industry.

The rest of this article follows: The second part is the literature review and research hypothesis, the third part is selection of data indicators and the establishment of models, the fourth part is the empirical results and discussion of empirical results, the fifth part proposed the policy suggestion according to the empirical conclusion.

## Literature review and research hypothesis

### Literature review

In the field of pharmaceutical manufacturing industry, most scholars focus on the research of high-tech industry as a whole, but less on the research of pharmaceutical manufacturing industry in the field of high-tech industry^13,14^. Moreover, for the efficiency of pharmaceutical manufacturing industry, most scholars focus on innovation efficiency^2^, investment efficiency^15,16^ and enterprise management efficiency^17,18^, but there is less research on the environment of pharmaceutical manufacturing industry. Lee and Brorson^19^ believed that the pharmaceutical manufacturing industry is an important driving force for modernization, and innovative production technology helps to prevent the shortage of drugs in recent years^19^. Shi (2019) studied the diversified agglomeration, specialized agglomeration and innovation efficiency of pharmaceutical manufacturing industry. Specialized agglomeration is not conducive to the improvement of innovation efficiency of pharmaceutical manufacturing industry^20^. Li and Liu (2019) used the super-SBM model to measure the impact of relevant incentive policies on the innovation efficiency of high-end manufacturing industry in China from 2012 to 2017^21^. Cheng (2020) used the multiple linear regression model with risk analysis to study the impact of technical factors on energy saving potential and manufacturing transformation and upgrading^22^.

In the field of environmental regulation, scholars have carried out extensive and in-depth research, scholars based on different perspectives or with different research methods come to conclusions are also widely divergent. Wang et al. (2021) studied the impact of environmental regulation on the spatial spillover effect of regional innovation, and concluded that innovation output, environmental regulation and R&D internal expenditure are innovation spillovers^23^. Pan et al. (2021) studied the impact of environmental regulatory policy on cleaner production technology innovation, and used regional pollution intensity and R&D investment scale explain the heterogeneity effect between them^24^. Based on the panel data of manufacturing enterprises, Li et al. (2021) analyzed the impact of environmental regulation on the efficiency of technological innovation in China's manufacturing industry from the regional, industrial and enterprise levels^25^. Zhou et al. (2021) used that spatial capacity model to study the impact of environmental regulation on cities, and thought that environmental regulation has a significant positive impact on urban innovation^26^. The impact of environmental regulation on the efficiency can be divided into three main types: promoting^27–29^, inhibiting^25,30^ and "U" shaped relationship^31^. The reasons for the controversy over the direction of environmental regulation influence mainly include different research areas, different measurement methods, different calculation methods of environmental regulation and different variables. The existing results provide a good reference value for this paper. In this paper, the square item of environmental regulation will be added to study whether environmental regulation has a U-shaped impact on the total factor productivity of pharmaceutical manufacturing industry. And then judge the direction of environmental regulation impact.

In the study of the impact of environmental regulation on pharmaceutical manufacturing industry, most scholars focus on the impact of environmental regulation on high-tech industry or industrial industry, and the model construction and analysis point of view are also different. Zhang (2021) used non-radial SBM model to measure the industrial green efficiency of each province, and then made Tobit regression on environmental regulation and green efficiency^32^. Based on the SBM model and the panel Tobit model, Yi et al. (2020) studied the impact of government R&D subsidies and environmental regulation on the green innovation efficiency of manufacturing industry in the Yangtze River Economic Zone^33^. Qiu et al. (2021) used feasible generalized least squares (FGLS) and dynamic generalized method of moments (GMM) to study the impact of environmental regulation and foreign direct investment on green total factor productivity of industrial sectors in 30 provinces of China^34^. Similarly, Xu et al. (2021) also studied the impact of environmental regulation and foreign direct investment on China's green total factor productivity^35^. By establishing a panel Poisson fixed effect model, Cai et al. (2020) studied the incentive effect of environmental regulations on green technology innovation of listed companies in heavy pollution industries in China^36^. Zhao et al. (2021) used SYS-GMM and DIF-GMM to study the impact of environmental regulation and technological innovation on the green transformation of manufacturing industry in the Yangtze River Economic Zone^37^.

Based on the above analysis, this paper will make the following innovations in the existing research: First, in terms of theory, this paper will study the direct effect and spatial spillover effect of environmental regulation on total factor productivity of pharmaceutical manufacturing industry, which enriches the research results in the field of cleaning in pharmaceutical manufacturing industry; Second, this paper constructs the Metafrontier Malmquist-Luenberger index which considers the undesired output mixed distance EBM model to measure the total factor productivity of pharmaceutical manufacturing industry. Compared with the DEA model without unexpected output and the single distance function model, the calculation results of the model in this paper are more accurate and effective; Third, this paper considers the mediating effect of environmental regulation on pharmaceutical manufacturing industry from three aspects of green technology, production technology and industrial structure, and considers the heterogeneity of eastern, central and western regions, then comprehensively analyzes the impact of environmental regulation on pharmaceutical manufacturing total factor productivity.

### Research hypothesis

Generally speaking, high-tech industry is considered to be a pollution-free and efficient industry, especially its knowledge-intensive and technologically advanced characteristics^38,39^. However, it cannot be ignored that there is a certain waste of resources in the high-tech industry from the perspective of production mode, and its production mode is still dominated by "traditional resources-input-consumption-waste discharge"^19^. In 2020, Ministry of Ecological Environment of the People’s Republic of China issued the Second National Pollution Source Census Bulletin. In the ranking of industrial water pollution, the pharmaceutical manufacturing industry is in the forefront. The COD and BOD5 of pharmaceutical manufacturing enterprises are as high as tens of thousands or even hundreds of thousands^40^. When wastewater enters the water body, it will consume dissolved oxygen in the water. In addition, when wastewater enters the water body, it will produce high concentrations of cyanogen, phenol, antibiotics and so on, which are difficult to degrade and have biological toxicity^41^. At the same time, the chemical reaction of pharmaceutical manufacturing industry is often accompanied by inorganic waste gas, organic waste gas, chemical comprehensive waste gas and other emissions. These waste gases are complex, difficult to collect and control, and may even be inhaled through breathing and skin, endangering health^42^. Therefore, the pharmaceutical manufacturing industry has a problem of environmental pollution that cannot be ignored.

As the environment as a kind of public goods with externalities, often rely on the role of government to guide. Environmental regulation is an important tool for effectively control of environmental pollution, which will achieve green transformation of pharmaceutical manufacturing industry, and improve production efficiency^37^.

#### The spatial spillover effect of total factor productivity in pharmaceutical manufacturing industry

According to Tobler's First Law of Geography, everything is related to other things. It's just that things are more closely related. The geographical distance, the level of economic development and the degree of scientific and technological innovation in each region in space all affect the strength of environmental regulation, the level of total factor productivity of pharmaceutical manufacturing industry and the relationship between them. Anselin (1988) also pointed out that a region will be more or less affected by the surrounding areas^43^. Ignoring spatial correlation can lead to the underestimation of interregional spillover effect and external effect, which leads to the deviation of model setting and prediction results^44^.

Economic subjects often cannot exist independently. With the deepening of economic globalization, the interrelationship between regions and countries is becoming closer and closer. The development of a region will inevitably affect the development of surrounding areas. The development of total factor productivity in pharmaceutical manufacturing industry will also produce such a spatial effect.

Due to the differences in resource endowment and technological innovation, the total factor productivity of pharmaceutical manufacturing industry in different provinces presents different spatial distribution characteristics, which causes the flow of factors among provinces and affects the total factor productivity of pharmaceutical manufacturing industry in surrounding area.

From the perspective of resource endowment, most pharmaceutical manufacturing enterprises will choose areas rich in pharmaceutical resources to put into operation. According to the classical location theory, on the one hand, the cost of raw materials is relatively low in the areas rich in medical resources, and the selection of pharmaceutical manufacturing enterprises in this area can effectively reduce production costs. On the other hand, obtaining raw materials in areas rich in medical resources can reduce the transportation cost of raw materials and ensure the freshness of raw materials. The location of pharmaceutical manufacturing industry will affect the location of R & D, logistics and other supporting service enterprises in the downstream of the industrial chain, which is very important for the formation of pharmaceutical manufacturing industry base in the region. As an important medicinal material producing area in northern China, Changbai Mountain in Jilin Province occupies an important position in the production of pharmaceutical manufacturing industry, which promotes the development of pharmaceutical manufacturing industry in Northeast China. Guangdong and Guangxi are located in the subtropical region of South China, with up to 5000 species of medicinal plants and only 300 species of medicinal animals, rich in animal and plant medical resources. Resource endowment is an important reason for the formation of regional differences.

From the perspective of technological innovation, pharmaceutical manufacturing industry, as a subdivision of high-tech industry, needs to have a higher scientific and technological content. Areas with high technology innovation content can share the results of technology spillover, upgrade technology quickly and reduce the cost of enterprise innovation by re-imitating and re-innovating. In addition, the places with higher scientific and technological content generally have higher human capital, which provides better talent support for promoting the development of pharmaceutical manufacturing industry. For example, under the background of the strong combination of medical schools, pharmaceutical enterprises, high-level universities, pharmaceutical research institutes and clinical hospitals jointly rely on the advantages of regional science and technology and talents. It reduces the cost of innovation and improves the conversion rate of innovation achievements. Therefore, the content of regional technological innovation will directly affect the differences in the spatial distribution of pharmaceutical manufacturing industry.

**Figure 1:**
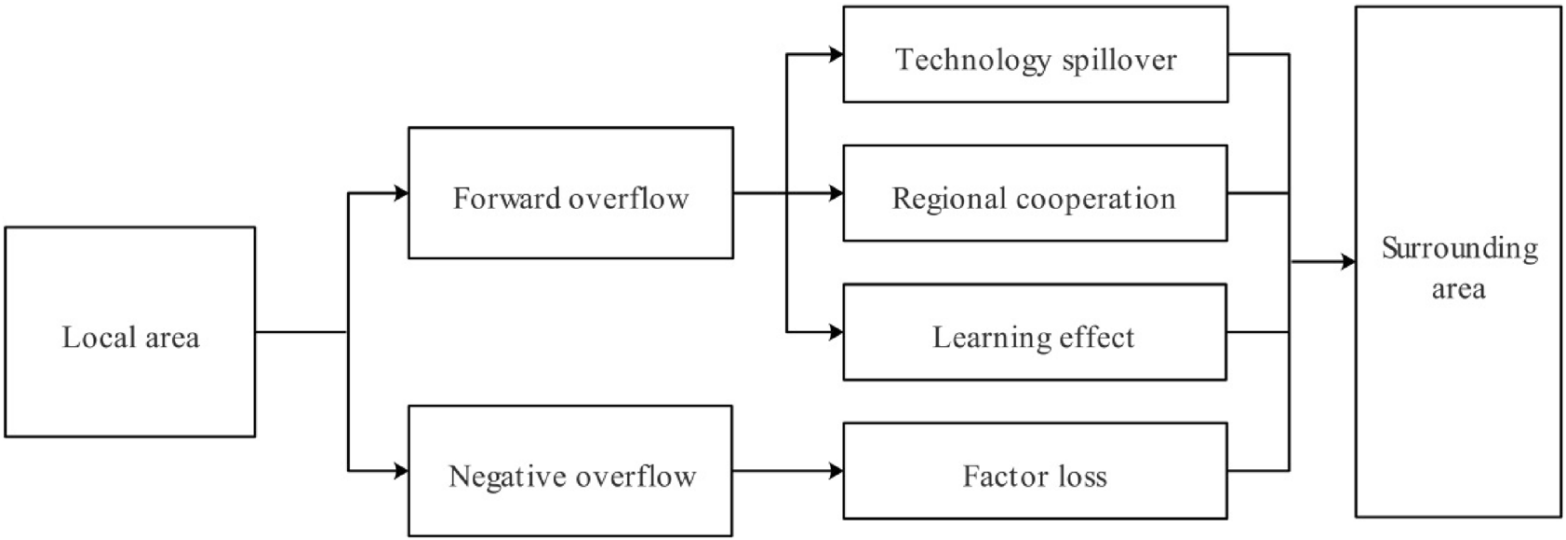
The spatial spillover effect of HTFP.

The differences in the spatial distribution of pharmaceutical manufacturing industry in different provinces lead to the spillover effect of total factors of pharmaceutical manufacturing industry in the region to the surrounding areas. According to the total factor productivity of the pharmaceutical manufacturing industry in the region led to changes in the total factor productivity of the surrounding areas, can be divided into positive spillover and negative spillover (Fig. 1).

Among them, the positive spillover through technology spillover, regional cooperation and learning effect performance.

From the perspective of technology spillover, as a subdivision of high-tech industry, pharmaceutical manufacturing industry has the characteristics of high technology content of products. The innovation activities of pharmaceutical manufacturing industry increase the total amount of knowledge available to the whole society, and bring positive externalities of knowledge to all pharmaceutical manufacturing industries. On the one hand, the part of innovation activities obtained without threshold, such as published papers, directly improves the knowledge reserve of pharmaceutical manufacturing industry in other regions. This is the most obvious manifestation of knowledge spillover effect. On the other hand, although the achievements protected by property rights in pharmaceutical manufacturing industry, such as invention patents, cannot be directly used by other pharmaceutical manufacturing enterprises, such achievements expand the total amount of technology and knowledge reserve owned by the whole society, and have a positive spillover effect on the pharmaceutical manufacturing industry in the surrounding areas through technology trade, patent transfer and talent transfer.

From the perspective of regional cooperation, regional resource endowment, industrial division of labor, historical process and other aspects will affect regional cooperation. The regions where the pharmaceutical manufacturing industry is relatively concentrated (such as the Yangtze River Delta urban agglomeration and the Pearl River Delta urban agglomeration) have the advantages of convenient sea, land and air transportation in geographical features, and have formed the characteristics of undertaking the core departments by the central cities (such as Shanghai, Nanjing, Guangzhou and Shenzhen), sharing the manufacturing factories by the surrounding cities and upstream industries. The cross-regional division of labor in the industrial chain promotes the flow of technology between regions, and the departments that master advanced technology and core knowledge transfer knowledge and advanced management capabilities to other departments through upstream and downstream cooperation and exchange, product design and production, thus producing positive spatial spillover effects.

From the perspective of learning effect, when the local pharmaceutical manufacturing industry performs well in technological innovation, organizational management, clean production and other aspects, and then makes great achievements in profitability, market position and other aspects, it will inevitably have an impact on the pharmaceutical manufacturing industry in the surrounding areas. This kind of influence may arise with the news media and public opinion, as well as with the exchanges between enterprises and governments. This influence will promote the learning behavior of the surrounding areas. In order to pursue economic interests and sustainable development, the surrounding areas will imitate the excellent cases of the region, that is, the learning effect, thus producing a positive spatial spillover effect.

For example, in Jiangsu, Zhejiang and Shanghai, the geographical location of the three provinces is relatively close, and there are great similarities in economic development, factor allocation, technological innovation, talent construction and other fields. A positive spatial effect has been formed between regions, and the total factor productivity of pharmaceutical manufacturing industry has been greatly improved. In 2017, Shanghai Pharmaceutical, East China Pharmaceutical and Fuxing Pharmaceutical, as the leading A-share listed pharmaceutical enterprises in Jiangsu, Zhejiang and Shanghai, had a total of over 50 million varieties. Medicine is one of the important pillar industries in Jiangsu, Zhejiang and Shanghai. Pharmaceutical manufacturing industry has become one of the important pillar industries in Jiangsu, Zhejiang and Shanghai.

Negative spillover is mainly reflected in the loss of elements. In space, when the "highland" of the development of pharmaceutical manufacturing industry is surrounded by multiple "depressions", resulting in a "dominant" situation, the degree of production factors gathering from the surrounding areas to the center will be very obvious. When more and more high-tech talents and capital gather from the surrounding areas to the central area with convenient traffic conditions, they provide sufficient support for the total factor productivity of the pharmaceutical manufacturing industry in the central area, at the same time, the surrounding areas gradually lose the development power of the pharmaceutical manufacturing industry due to the continuous outflow of talents and capital, and it is difficult to maintain the development of the pharmaceutical manufacturing industry. In this case, there is a negative spatial spillover effect.

For example, in the Beijing-Tianjin-Hebei region, compared with Beijing and Tianjin, Hebei has disadvantages that cannot be ignored in both economic development and talent base. This situation will lead to Beijing and Tianjin absorbing a large number of resource elements in Hebei, resulting in brain drain and capital loss in Hebei, which makes the total factor productivity of Hebei's pharmaceutical manufacturing industry low. However, in 2020, the Beijing-Tianjin-Hebei region has initially formed an industrial development pattern with Beijing as the core, Tianjin and Hebei complementary cooperation, showing a spatial cluster development model. In order to actively undertake the transfer of biomedicine industry, which is now known at home and abroad, and has formed Shijiazhuang, Hebei Province, Cangzhou and Anguo are three major industrial bases. The inhibitory effect of factor loss is also further reduced.

#### The direct impact of environmental regulation on total factor productivity of pharmaceutical manufacturing industry

**Figure 2:**
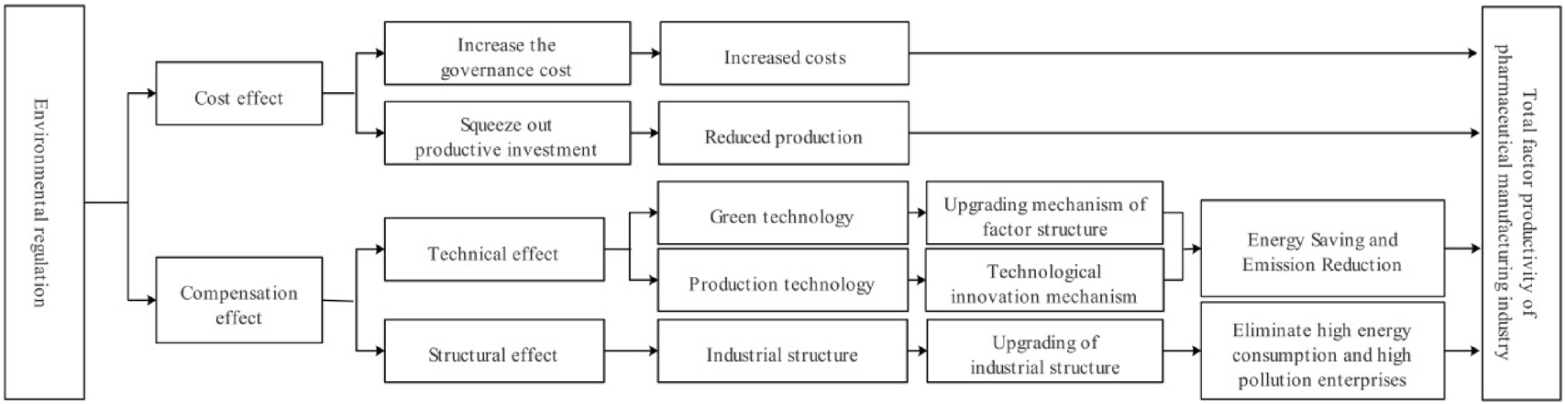
The direct impact of ER on HTFP.

The direct impact of environmental regulation on the total factor productivity of pharmaceutical manufacturing industry is shown in Fig. 2. It can be divided into cost effect and compensation effect^45^. Among them, in the cost effect, environmental regulation has a inhibitory effect on the total factor productivity of pharmaceutical manufacturing industry, while compensation effect promotes it.

First, the improvement of environmental regulation standards will increase the production costs of pharmaceutical manufacturing enterprises. Because the improvement of environmental regulation standards means that pollution emissions need to be controlled within the scope of standards. Enterprises are forced to use part of the funds to pay pollution taxes and fees and carry out pollution prevention and control, thus increasing the production costs of enterprises^46–48^. The increase of production cost will occupy the productive investment of enterprises and reduce the production of enterprises, thus inhibiting the total factor productivity of pharmaceutical manufacturing industry.

But at the same time, the improvement of environmental regulation intensity can play a compensatory role. According to Porter's hypothesis, good environmental regulation will play a better incentive and guidance role in the production mode of enterprises. Enterprises increase R & D investment through innovation compensation effect, thus promoting the total factor productivity of pharmaceutical manufacturing industry. The compensation effect can be reflected by the technical effect and the structural effect, in which the technical effect can be divided into green technology and production technology^49,50^.

According to the upgrading mechanism of factor structure, the improvement of environmental regulation intensity will inevitably increase the price of non-renewable energy such as coal and oil, thus reducing the use of non-renewable energy. This situation will enable enterprises to find cleaner energy production, thus improving the total factor productivity of pharmaceutical manufacturing industry.

According to the mechanism of technological innovation, "Porter Hypothesis" holds that with the improvement of environmental regulation intensity, enterprises will increase the cost of pollution control, thus reducing the profits of enterprises. In order to compensate for this part of profits, enterprises will increase the demand for environmental protection products, which will force enterprises to increase investment in scientific research and improve technological innovation. Improving technological innovation plays an important role in improving production efficiency and improving the competitiveness of enterprises in the market, at the same time, the government will introduce a series of subsidies and tax reduction policies to encourage enterprises to carry out environmental protection technological innovation through incentive environmental regulations. Thus in this incentive environment regulation policy, the enterprise environment; Infection has been effectively controlled and output has been increased, thus improving the total factor productivity of pharmaceutical manufacturing industry.

According to the above cost effect theory, the improvement of environmental regulation will increase the cost of high energy-consuming enterprises, thus strictly controlling the entry of resource-consuming and pollution-intensive enterprises. According to the theory of industrial structure upgrading, cost increase will force enterprises to increase investment in research and promote the transformation of high energy-consuming enterprises to clean enterprises. Enterprises that cannot complete the improvement and upgrading in time will inevitably face elimination, thus speeding up the withdrawal of such enterprises and promoting the upgrading of the pharmaceutical manufacturing industry. In addition, the increase of environmental regulation intensity will increase the cost of enterprises. In order to reduce costs, enterprises will choose to unite enterprises, use production equipment together, maintain pollution treatment results together, and form scale remuneration for pollution treatment, which promotes industrial agglomeration, promotes production exchanges within and between regions, and further improves the total factor productivity of pharmaceutical manufacturing industry^27^.

In the process of practice, Yuekang Pharmaceutical Group Co., Ltd. has formed a pharmaceutical industry chain development pattern based on Anhui API base and four characteristic preparation bases in Beijing, Shanghai, Guangzhou and Chongqing. It has established seven environmental strategic topics to guide environmental protection in enterprise operation, and upgraded the green industry in R & D, procurement, logistics, production process and products. It aims to build a green factory with environmental affinity and create an enterprise with sustainable development ability and core competitiveness through continuous environmental improvement.

#### The indirect impact of environmental regulation on the total factor productivity of pharmaceutical manufacturing industry

**Figure 3:**
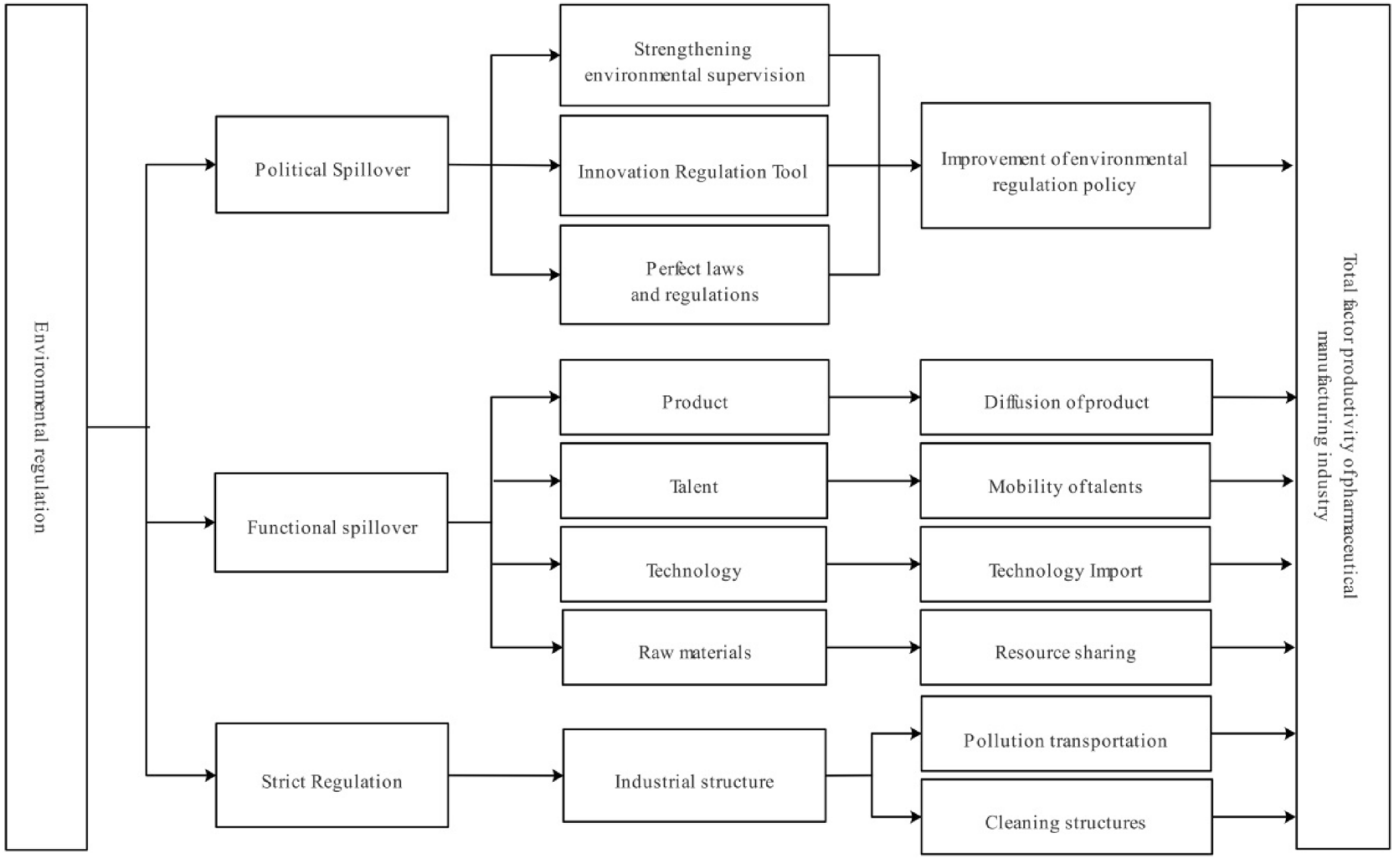
The indirect impact of ER on HTFP.

The direct impact of environmental regulation on the total factor productivity of pharmaceutical manufacturing industry is shown in Fig. 3. In the process of the spatial impact of local environmental regulation on the total factor productivity of pharmaceutical manufacturing industry in the surrounding areas, it is mainly based on the two transmission mechanisms put forward by the new functionalism theory. It shows the spatial spillover effect of environmental regulation.

Mccormic (1999)’s "spillover" to the theory of neo-functionalism is summarized as political spillover and functional spillover^51^. Political spillover refers to the reform of economic activities and decision-making in the political field by learning innovative policies of neighboring countries or regions in the process of integration. Functional spillover refers to cooperation and exchanges in the relevant fields of integration construction, so as to spill resources in specific fields to relevant departments or regions. In the spatial effect brought by environmental regulation, there are both political spillovers and functional spillovers. In addition, the spatial effect of environmental regulation also has the barrier effect brought by the cost effect to the surrounding areas.

From the perspective of political spillover, the local environmental regulation policy has been used for reference by the governments of the surrounding areas through conference exchanges, document learning, investigation and other spillover channels. In order to optimize the environmental regulation policies of the surrounding areas, the institutional diffusion between regions has been formed. The governments of the surrounding areas have integrated the lessons learned into the local environmental regulation management, optimized the establishment of laws and regulations, strengthened the supervision of environmental regulation, and innovated the tools of environmental regulation. Thus, the environmental regulation policy in this area has been improved. Through the direct impact of environmental regulation, it promotes the improvement of total factor productivity of pharmaceutical manufacturing industry.

From the perspective of functional spillover, the production performance caused by environmental regulation in a region will bring spillover results to the surrounding areas through learning effect and demonstration effect. Through the channels of product diffusion, talent flow, technology introduction and resource sharing, the surrounding areas have absorbed advanced management experience and product innovation achievements in the fields of products, talents, technology and raw materials. The positive external effect of local environmental regulation on the total factor productivity of pharmaceutical manufacturing industry in surrounding areas has been formed.

However, from the perspective of the cost effect brought by environmental regulation, environmental regulation has increased the local production cost. At this time, enterprises can choose to transfer, so as to avoid high emission reduction costs^52^. These high energy-consuming enterprises will consider the cost of technological innovation and the cost of enterprise transfer, when the cost of technological innovation is greater than the cost of transfer, these enterprises will transfer to the surrounding areas. In the formation of large-scale industrial transfer, the industrial structure of the surrounding areas will change, and will bear the negative environmental externalities brought by these energy-consuming enterprises. Therefore, in this case, the improvement of local environmental regulation will have a negative spatial spillover effect on the total factor productivity of pharmaceutical manufacturing industry in surrounding areas.

For the cost effect of business migration, enterprises will indeed consider the cost when they first set up enterprises. However, it is worth noting that when the initial establishment of enterprises, the environmental regulation at that time was considered (assuming that the decision-making of enterprises is rational). But environmental regulation changes with time. In other cases, enterprises will re-select the location according to the intensity of environmental regulation. If the cost of technological transformation is greater than the cost of migration, the enterprise may choose to migrate to the surrounding provinces.

In the process of practice, Beijing, as the capital of China, has a strong government incentive environmental regulation policy. However, considering the high cost of technological innovation in Beijing, some enterprises will choose to migrate to Hebei, which is close to Beijing, for production because their financial strength cannot support the high cost of innovation. Hebei's innovation cost is low, and has a relatively weak environmental regulation policy, so some enterprises in Beijing migrating to Hebei is a better strategy. However, this strategy has brought barriers to the development of Hebei's pharmaceutical manufacturing industry. However, in recent years, in the Beijing-Tianjin-Hebei region, with Beijing as the core, Tianjin and Hebei complementary and cooperative industrial development pattern, showing a spatial cluster development model. The intensity of environmental regulation in Hebei region has been continuously improved, and the threshold and cost of regional migration of high energy-consuming enterprises have been raised, which effectively limits the negative spatial spillover effect brought by cost effect. Based on the deepening of the cluster development model, the positive spatial spillover effect brought by politics and functionality is constantly improving. Therefore, on the whole, the improvement of environmental regulation will have a positive spatial spillover effect on Hebei.

However, it is worth noting that as a kind of policy regulation, the strength of environmental regulation will be limited by social cleanliness goals, green transformation process, pollution treatment performance and other factors. So the environmental regulation cannot be increased indefinitely in practice. Therefore, the impact of environmental regulation on total factor productivity of pharmaceutical manufacturing industry discussed in this paper is based on the impact before environmental regulation has not reached the limit. The subsequent relevant assumptions and conclusions are also based on the above facts. To sum up, according to the above theory and practical analysis, this paper puts forward the following assumptions.

According to the direct effect of environmental regulation on total factor productivity of pharmaceutical manufacturing industry, this paper puts forward Hypothesis 1: Before the environmental regulation (ER) reaches the limit, environmental regulation has a significant role in promoting the pharmaceutical manufacturing total factor productivity (HTFP).

According to the indirect effect of environmental regulation on total factor productivity of pharmaceutical manufacturing industry, this paper puts forward Hypothesis 2: Before reaching the limit, environmental regulation (ER) will have a significant role in promoting the total factor productivity of the pharmaceutical manufacturing industry (HTFP) in the surrounding areas, and at the same time, the improvement of pharmaceutical manufacturing total factor productivity in the surrounding areas will also promote the improvement of pharmaceutical manufacturing total factor productivity in the region.

According to the intermediary effect of green technology, production technology and industrial structure, this paper puts forward Hypothesis 3: Green technology, production technology and industrial structure play an intermediary role in the impact of environmental regulation (ER) on total factor productivity of pharmaceutical manufacturing industry (HTFP).

## Method and data

This part is the selection of data indicators and the establishment of models. “Indicator s election” section refers to the selection of data indicators. “Measurement of pharmaceutical manufacturing total factor productivity” section is the calculation process of HTFP. “Spatial econometric model” section is the establishment of spatial econometric model.

### Indicator selection

This paper studies the effect of environmental regulation on total factor productivity of pharmaceutical manufacturing industry. According to the availability and accuracy, we select the panel data of 30 provinces in China except Hong Kong, Macao, Taiwan and Tibet from 2004 to 2019 to study. In the heterogeneity analysis, 30 provinces in China are divided into eastern, central and western regions^53,54^, with Beijing, Tianjin, Hebei, Liaoning, Shanghai, Jiangsu, Zhejiang, Fujian, Shandong, Guangdong and Hainan in the eastern region, Shanxi, Jilin, Heilongjiang, Anhui, Jiangxi, Henan, Hubei and Hunan in the central region and Inner Mongolia, Guangxi, Chongqing, Sichuan, Guizhou, Yunnan, Shaanxi, Gansu, Qinghai, Ningxia and Xinjiang in the western region.

The data selected in this paper are all from *China Statistical Yearbook*, *China Environmental Yearbook*, *China High-tech Industry Statistical Yearbook*, *China Environmental Statistical Yearbook*, *China Industrial Statistical Yearbooks*, *China Health Statistical Yearbooks* and provincial statistical yearbooks.

This paper takes the logarithm of environmental regulation (ER) as the core explanatory variable, and the logarithm of total factor productivity of pharmaceutical manufacturing industry (HTFP) as the dependent variable.

The connotation of environmental regulation (ER) varies with the research focus. However, at the present stage of research, most of the connotations of environmental regulation have a certain degree of convergence. The main body of environmental regulation is the government and social public institutions, and the object is enterprises or individuals. The purpose of implementing environmental regulation is to reduce the negative externalities of pollution and improve environmental performance. Therefore, in this paper, environmental regulation will adopt the concept generally defined by the academic circles: in order to ensure the sustainable development of resources and environment, the government takes certain measures against the micro-subjects. It makes the production mode and decision-making of micro-individuals change, thus affecting the development of market resource allocation in the direction of green and sustainable development, and reduces the negative externality of environmental pollution, which achieves coordinated development of environmental protection and economic growth.

**Figure 4:**
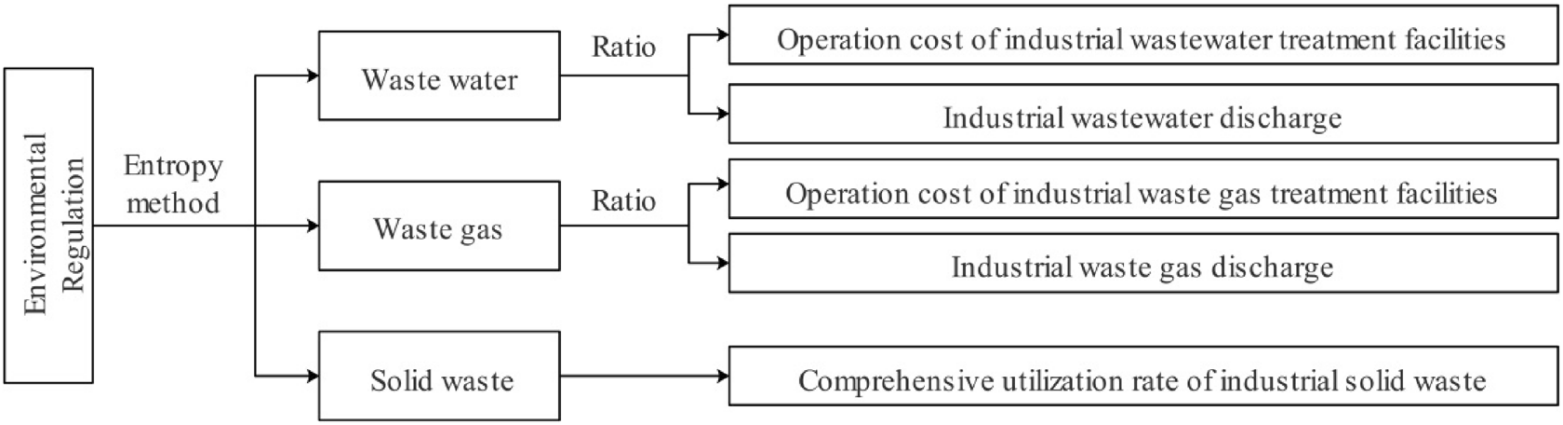
Measurement of environmental regulation.

As for the measurement of environmental regulation, at this stage, the academic circles mostly consider from the perspective of pollution control investment and effectiveness. The main measurement methods are the following three categories: First, through the governance input data to reflect environmental regulation. Investment in pollution control refers to the cost spent by the government or enterprises on pollution control. The more the input cost, the stronger the environmental regulation. Second, reflect environmental regulation through pollutant emission data. Pollutant emissions are mainly measured by the pollution emissions generated by the total output value per unit of the industry or the output value per unit of sales. The more pollutants are discharged, the weaker the degree of environmental regulation is. Third, by building a comprehensive index of multiple environmental indicators to measure the environment system. In this paper, we will consider the investment of pollution control and the effectiveness of pollution control, and build a comprehensive index containing a variety of pollutants to measure environmental regulation (Fig. 4). According to Lenson (1996) and Yuan and Chen (2014), the ratio of operating cost of industrial wastewater treatment facilities to industrial wastewater discharge, The entropy weight method^55^ is used to obtain environmental regulation (ER) for the ratio of industrial waste operation cost to industrial waste discharge and the comprehensive utilization rate of industrial solid waste^56,57^.

The total factor productivity of pharmaceutical manufacturing industry (HTFP) is measured by data envelopment analysis method. The Metafrontier Malmquist-Luenberger index based on the mixed distance EBM model is constructed to measure the pharmaceutical manufacturing total factor productivity from 2004 to 2019. See “Measurement of pharmaceutical manufacturing total factor productivity” section for the specific calculation process.

The control variables selected in this paper are the degree of foreign development (*Open*), the level of labor force (*Labor*), assets (*Capital*), operating conditions (*Income*) and profitability (*Profit*). Among them, the degree of opening to the outside world is expressed by the 1/100,000 of total import and export volume of goods of foreign-funded enterprises. The level of labor force is expressed by the 1/10 of average number of employees in industries above scale. Assets, operating conditions and profitability were the 1/10 of total assets of industries above scale, the main business income and total profits to show.

This paper considers the intermediary conduction mechanism from the technical effect and the structure effect, the technical effect subdivides into the green technology and production technology. The green technology (*Ingrva*) is represented by a value of the number of percentage of green utility models in the total number of utility models applied annually in the region. Production technology (*RD*) is expressed as a value 1/10 of the R&D personnel. The industrial structure (*Structure*) is expressed by the value of the ratio of the sales output value of the pharmaceutical manufacturing industry.

The descriptive statistical analysis of the variables selected in this paper is shown in Table 1.

**Table 1:** Descriptive statistical analysis of each variable.

Variable	Obs	Mean	Std. Dev	Min	Max
HTFP	480	410.917	432.2287	56.04605	4905.364
ER	480	8.953651	5.276614	1.245677	54.79272
Open	480	51.71258	106.1336	0.004147	592.0705
Labour	480	27.68299	29.94936	0.928	156.8
Capital	480	514.961	820.0662	1.102	4597.224
Income	480	625.2461	1053.965	1.176	5262.673
Profit	480	41.59898	68.6194	0.1	372.316
Ingrva	480	108.7019	58.24322	7.61	279.48
RD	480	280.8496	371.0577	0.6	1858.8
Structure	480	572.2567	839.702	3.26	6156.78

### Measurement of pharmaceutical manufacturing total factor productivity

This paper constructs the Metafrontier Malmquist-Luenberger index under the mixed distance EBM model to measure the total factor productivity of pharmaceutical manufacturing industry. Before measuring, we need to build an input–output index system. In this paper, we will take the average number of workers and health technicians in pharmaceutical manufacturing industry as labor input indicators^58–60^, the number of beds and enterprises as capital input^61^, the cost of inpatients and outpatients as treatment support input^62^, business income and profit as expected output^63^, and the incidence and mortality of category A and B infectious diseases as unexpected output^33^. The construction of medical efficiency system in academia provides a good idea for the construction of medical total factor productivity index system in this paper. In order to construct the index system of total factor productivity in pharmaceutical manufacturing industry. And that construct system is shown as Table 2.

**Table 2:** Input–output index system.

Level I indicators	Level II indicators	Level III indicators
Input indicators	Labor input	Annual average employees
		Number of health technicians per thousand population
	Infrastructure input	Number of beds in medical and health institutions
	Scale input	Number of enterprises
	Guarantee input	Per capita medical expenses of outpatients
		Per capita medical expenses of inpatients
Output indicators	Expected output	Revenue
		Profits
	Unexpected output	Incidence of category A and B infectious diseases (1/100,000)
		Mortality rate of category A and B infectious diseases (1/100,000)

The hybrid model (EBM) proposed by Tone and Tsutsui (2010) took into account the radial and non-radial distance functions, and used to effectively solve the limitations of the traditional DEA model^64^. They proposed an EBM model on the basis of fully considering the ratio of radial and non-radial relaxation, which fully considers the differences of relaxation variables of various elements. Based on the assumption of CRS (constant scale return), the expression of the initial oriented EBM model is:

$$\begin{gathered} \lambda^{*} = \min \theta - \varepsilon_{x} \sum\limits_{p = 1}^{P} {\frac{{w_{p}^{ - } s_{p}^{ - } }}{{x_{pk} }}} \hfill \\ s.t. \sum\limits_{j = 1}^{J} {x_{pj} \delta_{j} + s_{p}^{ - } = \theta x_{pk} } ,p = 1, \ldots ,P \hfill \\ \sum\limits_{j = 1}^{J} {y_{qj} } \delta_{j} \ge y_{qk} ,q = 1, \ldots ,Q \hfill \\ \delta_{j} \ge 0,s_{p}^{ - } \ge 0 \hfill \\ \end{gathered}$$

where $x_{pj}$ and $y_{qj}$ are the inputs and outputs of decision unit k, respectively. *P* and *Q* are the amounts of inputs and outputs. There are 30 decision-making units (DMUs), namely 30 provinces in China except Hong Kong, Macao, Taiwan and Tibet.

The initial model is improved to a non-directed EBM model. The input-oriented initial EBM model does not include the programming solution of output, so the output constraint after the radial improvement parameters is added.

$$\sum\limits_{j = 1}^{J} {y_{qj} \delta_{j} - s_{q}^{ + } } = \varphi y_{qk} ,q = 1, \ldots ,Q$$

In the formula, $s_{q}^{ + }$ is the output relaxation variable, $\varphi$ is the output improvement parameter.

In order to further consider the importance of non-radial and radial in output planning, the weight difference of each output factor is analyzed concretely, and the constraint condition of fractional whole is considered, the radial and non-radial inefficiency is expressed in the form of summation. Therefore, the denominator part of the objective function can be expressed as:

$$\varphi + \varepsilon_{y} \sum\limits_{q = 1}^{Q} {\frac{{w_{q}^{ + } s_{q}^{ + } }}{{y_{qk} }}}$$

where $\varphi$ is the planning parameter of the radial part of the output index, $\varepsilon_{y}$ represents the importance of the non-radial part of the output index in the efficiency value, $w_{q}^{ + }$ represents the relative importance of the output index, and $\sum\limits_{p = 1}^{Q} {w_{q}^{ + } = 1}$.

So the undirected EBM model can be expressed as:

$$\begin{gathered} \lambda^{*} = \min \frac{{\theta - \varepsilon_{x} \sum\limits_{p = 1}^{P} {\frac{{w_{p}^{ - } s_{p}^{ - } }}{{x_{pk} }}} }}{{\varphi + \varepsilon_{y} \sum\limits_{q = 1}^{Q} {\frac{{w_{q}^{ + } s_{q}^{ + } }}{{y_{qk} }}} }} \hfill \\ s.t. \sum\limits_{j = 1}^{J} {x_{pj} \delta_{j} + s_{p}^{ - } = \theta x_{pk} } ,p = 1, \ldots ,P \hfill \\ \sum\limits_{j = 1}^{J} {y_{qj} \delta_{j} - s_{q}^{ + } } = \varphi y_{qk} ,q = 1, \ldots ,Q \hfill \\ \delta_{j} \ge 0,s_{p}^{ - } \ge 0,s_{q}^{ + } \ge 0 \hfill \\ \end{gathered}$$

The result $\lambda^{*}$ is between 0 and 1, the larger $\lambda^{*}$ is, the higher the efficiency of the DMU is. It indicates that the DMU is relatively efficient compared with others and is located on the production frontier.

Then the undesired outputs are added to the undirected EBM model. According to the method of Tone K(2001), this paper sets up the Undesirable-EBM^65^.

The constraints of the undesired output are:

$$\sum\limits_{j = 1}^{J} {b_{mj} \delta_{j} + s_{m}^{b - } = \varphi b_{mk} } ,m = 1, \ldots ,M$$

In that formula, $b_{mj}$ is the *m (m* = 1,…,*M)* unexpected output of the *k* decision making unit, and $s_{m}^{b - }$ is the unexpected output slack variable, and the unexpected output radial programming parameter is consistent with the expected output.

By adding additional weighted inefficiencies to the denominator of the objective function, the following results are obtained:

$$\varepsilon_{b} \sum\limits_{m = 1}^{M} {\frac{{w_{m}^{b - } s_{m}^{b - } }}{{b_{mk} }}}$$

In the formula, $\varepsilon_{b}$ represents the importance of the non-radial part of the non-expected output in the efficiency value, $w_{m}^{b - }$ represents the relative importance of each non-expected output index, and $\sum\limits_{m = 1}^{M} {w_{m}^{b - } = 1}$. So the objective function of the undirected EBM model of the undesired output can be expressed as:

$$\frac{{\theta - \varepsilon_{x} \sum\limits_{p = 1}^{P} {\frac{{w_{p}^{ - } s_{p}^{ - } }}{{x_{pk} }}} }}{{\varphi + \varepsilon_{y} \sum\limits_{q = 1}^{Q} {\frac{{w_{q}^{ + } s_{q}^{ + } }}{{y_{qk} }} + \varepsilon_{b} \sum\limits_{m = 1}^{M} {\frac{{w_{m}^{b - } s_{m}^{b - } }}{{b_{mk} }}} } }}$$

To sum up, the undirected EBM model considering the unexpected output is:

$$\begin{gathered} \lambda^{*} = \min \frac{{\theta - \varepsilon_{x} \sum\limits_{p = 1}^{P} {\frac{{w_{p}^{ - } s_{p}^{ - } }}{{x_{pk} }}} }}{{\varphi + \varepsilon_{y} \sum\limits_{q = 1}^{Q} {\frac{{w_{q}^{ + } s_{q}^{ + } }}{{y_{qk} }} + \varepsilon_{b} \sum\limits_{m = 1}^{M} {\frac{{w_{m}^{b - } s_{m}^{b - } }}{{b_{mk} }}} } }} \hfill \\ s.t. \sum\limits_{j = 1}^{J} {x_{pj} \delta_{j} + s_{p}^{ - } = \theta x_{pk} } ,p = 1, \ldots ,P \hfill \\ \sum\limits_{j = 1}^{J} {y_{qj} \delta_{j} - s_{q}^{ + } } = \varphi y_{qk} ,q = 1, \ldots ,Q \hfill \\ \sum\limits_{j = 1}^{J} {b_{mj} \delta_{j} + s_{m}^{b - } = \varphi b_{mk} } ,m = 1, \ldots ,M \hfill \\ \delta_{j} \ge 0,s_{p}^{ - } \ge 0,s_{q}^{ + } \ge 0,s_{m}^{b - } \ge 0 \hfill \\ \end{gathered}$$

According to Guo et al. (2017), the analysis framework of common frontier and group frontier can be constructed to study the total factor productivity of pharmaceutical manufacturing industry under different frontiers^66^, while this paper uses the efficiency value under common frontier as the basis of subsequent analysis, so here, only the model of measuring the efficiency value of common frontier is shown. According to Hayami and Ruttan (1971), the efficiency value can be obtained as follows^67^:

$$\begin{gathered} \min \alpha^{Metafrontier} = \frac{{\theta - \varepsilon_{x} \sum\limits_{p = 1}^{P} {\frac{{w_{p}^{ - } s_{p}^{ - } }}{{x_{pk} }}} }}{{\varphi + \varepsilon_{y} \sum\limits_{q = 1}^{Q} {\frac{{w_{q}^{ + } s_{q}^{ + } }}{{y_{qk} }} + \varepsilon_{b} \sum\limits_{m = 1}^{M} {\frac{{w_{m}^{b - } s_{m}^{b - } }}{{b_{mk} }}} } }} \hfill \\ s.t. \sum\limits_{j = 1}^{{J_{M} }} {x_{pj} \delta_{j} + s_{p}^{ - } = \theta x_{pk} } ,p = 1, \ldots ,P \hfill \\ \sum\limits_{j = 1}^{{J_{M} }} {y_{qj} \delta_{j} - s_{q}^{ + } } = \varphi y_{qk} ,q = 1, \ldots ,Q \hfill \\ \sum\limits_{j = 1}^{{J_{M} }} {b_{mj} \delta_{j} + s_{m}^{b - } = \varphi b_{mk} } ,m = 1, \ldots ,M \hfill \\ \delta_{j} \ge 0,s_{p}^{ - } \ge 0,s_{q}^{ + } \ge 0,s_{m}^{b - } \ge 0,j = 1, \ldots ,J_{M} \hfill \\ \end{gathered}$$

$J_{M}$ is the number of DMUs under the common front and $\delta$ is the intensity variable of the common front. According to Pastor and Lovell (2005), the Malmquist index can be constructed as follows^68^:

$$HTFP_{t}^{t + 1} = \sqrt {\frac{{1 - D_{t}^{m} (x^{t + 1} ,y^{t + 1} ,b^{t + 1} ;y^{t + 1} , - b^{t + 1} )}}{{1 - D_{t}^{m} (x^{t} ,y^{t} ,b^{t} ;y^{t} , - b^{t} )}} \times \frac{{1 - D_{t + 1}^{m} (x^{t + 1} ,y^{t + 1} ,b^{t + 1} ;y^{t + 1} , - b^{t + 1} )}}{{1 - D_{t}^{m} (x^{t} ,y^{t} ,b^{t} ;y^{t} , - b^{t} )}}}$$

$HTFP_{t}^{t + 1}$ means the total factor productivity of pharmaceutical manufacturing industry in the period from *t* to *t* + *1*, and $HTFP_{t}^{t + 1}$ is greater than 1, indicating that the total factor productivity of pharmaceutical manufacturing industry in that year is in an upward trend; $HTFP_{t}^{t + 1}$ is less than 1, indicating that the total factor productivity of pharmaceutical manufacturing industry is declining in that year. In order to ensure the validity of the follow-up measurement results, this paper uses the cumulative $HTFP_{t}^{t + 1}$ as the research basis, and its economic significance is the increase of total factor productivity of pharmaceutical manufacturing industry in period t relative to the base 2003.

The operation steps of the Max DEA software to realize the above model are as follows: Firstly, the data is imported into the Max DEA, and the corresponding indicators are selected for each variable. After the data is imported, the envelope model is selected, the EBM hybrid distance model is selected, the scale effect invariant option is selected, and the output indicator key “Incidence of category A and B infectious diseases (1/100,000)” and “Mortality rate of category A and B infectious diseases (1/100,000)” is placed in the model. The above step is the establishment process of the EBM model. The following will calculate the Metafrontier Malmquist-Luenberger, select the global reference index and select the common frontier model. From this, we can get the total factor productivity of pharmaceutical manufacturing industry under the common frontier.

### Spatial econometric model

Before the establishment of spatial econometrics, we need to establish a panel data regression model. Then we need to build a spatial weight matrix, use the spatial autocorrelation test, and we can establish a spatial econometrics model.

The panel data regression model is as follows:

$$HTFP_{it} = \beta + \alpha_{1} ERI_{it} + \rho X_{it} + \lambda z_{i} + \varepsilon_{it}$$

In the formula, $HTFP_{it}$ represents the value of the dependent variable in the *t* year of *i* province, *ER* is the core explanatory variable, and *X* is the control variable, $z$ represents the enterprise effect that does not change with time. $\varepsilon$ denotes a random perturbation term. $\alpha_{1}$ is the regression coefficient of the core explanatory variable *ER*, $\rho$ is the regression coefficient of the control variable, and $\beta$ is the constant coefficient.

The panel regression assumes that there is no difference among individuals, which is inconsistent with the actual situation. Therefore, considering the certain differences of economic subjects, individual effects can exist as fixed effects and random effects^69^.

The fixed effect model is as follows:

$$HTFP_{it} = \alpha_{2} ERI_{it} + \rho X_{it} + \lambda z_{i} { + }u_{i} + \varepsilon_{it}$$

The random effect model is as follows:

$$HTFP_{it} = \alpha_{3} ERI_{it} + \rho X_{it} + \lambda z_{i} { + }u_{i} + \varepsilon_{it}$$

Fixed effects and random effects are the same in the form of models, but random effects assume that $u_{i}$ is independent of $X_{it}$ and $z_{i}$, that is, individual effects are independent of explanatory variables, while fixed effects assume that u is dependent on at least one explanatory variable. So according to Hausman (1978), we need to introduce F test, LM test and Hausman test to determine whether there are individual effects, and whether individual effects are related to explanatory variables^70^.

In order to further discuss the existence of spatial autocorrelation, this paper uses Moran's I index to test the existence of spatial autocorrelation^71^. According to Cliff and Ord (1973), the global and local Moran's I index are formulated as follows^72^:

$$Global \,\,Moran^{\prime}s\,\, I = \frac{1}{{\sum\limits_{i = 1}^{n} {\sum\limits_{j = 1}^{n} {W_{ij} } } }} \times \frac{{n\sum\limits_{i = 1}^{n} {\sum\limits_{j = 1}^{n} {W_{ij} } } (x_{it} - \overline{x} )(x_{jt} - \overline{x} )}}{{\sum\limits_{i = 1}^{n} {(x_{it} - \overline{x} )^{2} } }}$$

$$Local \,\,Moran^{\prime}s \,\,I = \frac{{n(x_{it} - \overline{x} )\sum\limits_{j = 1}^{n} {W_{ij} } (x_{jt} - \overline{x} )}}{{\sum\limits_{i = 1}^{n} {(x_{it} - \overline{x} )^{2} } }}$$

where $x_{it}$ is the observed value of pharmaceutical manufacturing total factor productivity and ER in the year *t* of province *i*, and $W_{ij}$ is the spatial weight matrix. The global Moran index is between −1 and 1, with values greater than 0 indicating positive spatial autocorrelation and values less than 0 indicating negative spatial autocorrelation. When the local Moran index is greater than 0, it shows that there is "H–H" or "L-L" accumulation area in this area; When it is less than 0, it indicates that there is an "H–L" or "L–H" accumulation area in this area.

In this paper, 0–1 adjacency matrix (W1) and geographical distance matrix (W2) are used as the spatial weight matrix to test the spatial autocorrelation.W1 is used to establish the follow-up spatial econometric model, and W2 is used as the alternative weight matrix to test the robustness of the model.

According to You and Lv (2018), the 0–1 adjacency matrix (W1) is formulated as follows^73^:

$$W_{ij}^{1} = \left\{ {\begin{array}{*{20}l} {1,} \hfill & {adjacent} \hfill \\ {0,} \hfill & {other} \hfill \\ \end{array} } \right.$$

According to Wang et al. (2019), the geographical distance matrix (W2) is formulated as follows^74^:

$$W_{ij}^{2} = \frac{1}{{\left| {d_{ij} } \right|}}$$

where $d_{ij}$ is the distance between *i* province and *j* province.

On the basis of verifying the spatial autocorrelation of variables, this paper will establish a spatial econometric model to study the spatial spillover effect of ER on pharmaceutical manufacturing total factor productivity. In the existing research, spatial econometric models are mainly divided into spatial lag model (SAR), spatial error model (SEM) and spatial Durbin model (SDM). SAR only considers the spatial lag of the dependent variables, but does not consider the influence of the spatial lag of the explanatory variables^75^. SEM considers the case when the error term has spatial autocorrelation, but does not consider the effect of the spatial lag term of explanatory variables^76^. SDM takes into account both the dependent variable and the spatial lag of the explanatory variable, and the spatial spillover effect of the variable on the surrounding areas^77^.

The SAR model is as follows:

$$HTFP_{it} = \alpha_{4} ERI_{it} + \alpha_{5} W*HTFP_{it} + \rho X_{it} + \varepsilon_{it}$$

The SEM model is as follows:

$$\left\{ {\begin{array}{*{20}l} {HTFP_{it} = \alpha_{6} ERI_{it} + \rho X_{it} + \varepsilon_{it} } \hfill \\ {\varepsilon_{it} = \gamma W*\varepsilon_{it} + \nu_{it} } \hfill \\ \end{array} } \right.$$

The SDM model is the following:

$$HTFP_{it} = \alpha_{7} W*ERI_{it} + \alpha_{8} ERI_{it} + \alpha_{9} W*HTFP_{it} + \rho X_{it} + \delta W*X_{it} + \varepsilon_{it}$$

According to Elhorst (2012), this paper uses LR test to test the rationality of SDM model. LR test is to see whether SDM will degenerate into SAR and SEM models, which is to select the optimal spatial econometric model^78^.

## Empirical results and discussion

Based on the panel data of 30 provinces in China from 2004 to 2019, this paper studies the spatial spillover effects of environmental regulation (ER) on total factor productivity of pharmaceutical industry (HTFP) by establishing a spatial econometric model. “Temporal and spatial charact eri stics of ER and H TFP” section discusses the spatiotemporal characteristics of ER and pharmaceutical manufacturing total factor productivity. “Empirical results of panel data regression” section studies the effect of ER on pharmaceutical manufacturing total factor productivity through panel data regression. “Empirical results of spatial econometric model” section studies the spatial spillover effect of ER on pharmaceutical manufacturing total factor productivity through spatial Durbin model. “Further analysis” section further analyzes the intermediary transmission mechanism, regional heterogeneity, endogeneity and robustness of the two.

### Temporal and spatial characteristics of ER and HTFP

The pharmaceutical manufacturing total factor productivity index calculated by the Metafrontier Malmquist-Luenberger index under the mixed distance EBM model is the change rate from *t* to *t* + *1*, and has cumulative characteristics during the study period. Considering the accuracy of temporal-spatial analysis and the stability of the overall change, the annual average value of pharmaceutical manufacturing total factor productivity is used in “Temporal and spatial charact eri stics of ER and H TFP” section, and the cumulative value of pharmaceutical manufacturing total factor productivity is used in the subsequent measurement process.

**Figure 5:**
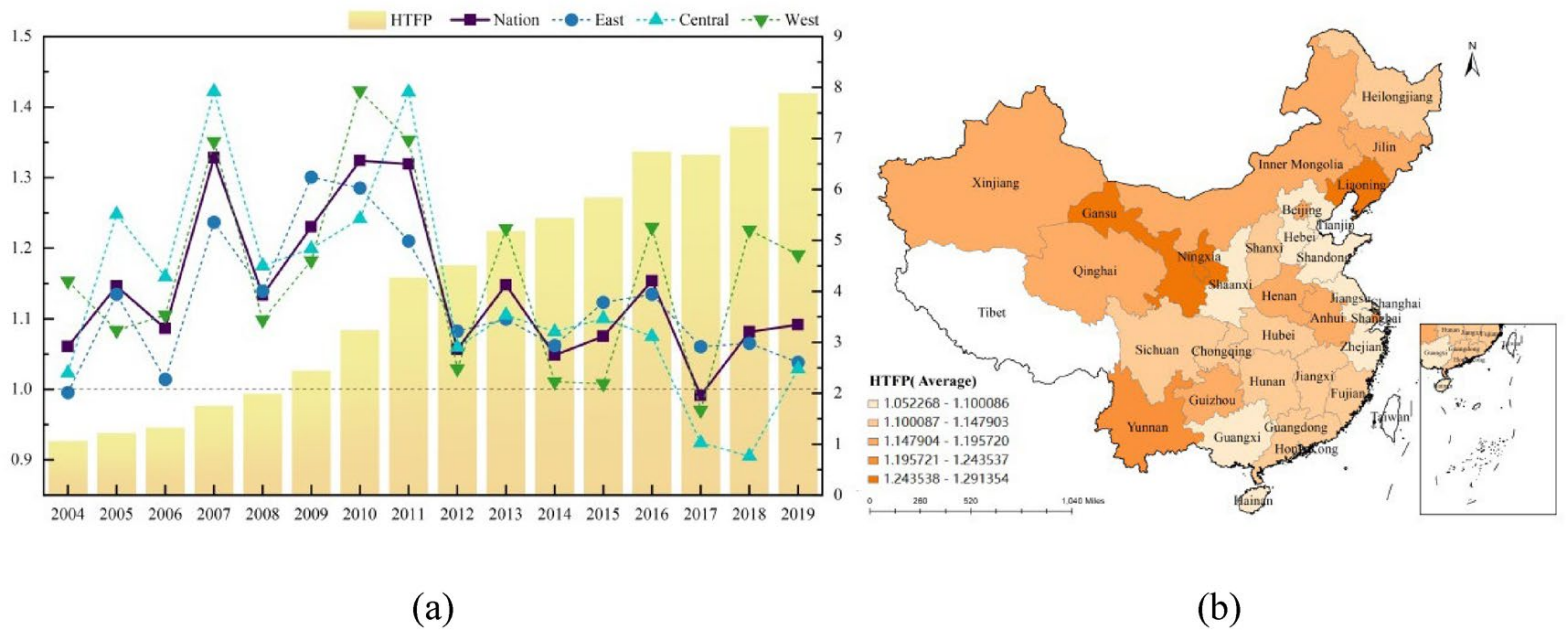
Temporal and spatial characteristics of HTFP.

Figure 5a describes the time trend of annual average change of pharmaceutical manufacturing total factor productivity and the change of cumulative value of pharmaceutical manufacturing total factor productivity in the whole country and the three major regions of east, middle and west. From the images, we can see that the average annual value of pharmaceutical manufacturing total factor productivity from 2004 to 2019 is greater than 1 except 2017, which indicates that pharmaceutical manufacturing total factor productivity is gradually increasing over time during the study period. In addition, pharmaceutical manufacturing total factor productivity reached the maximum (1.3241) in 2010, and then pharmaceutical manufacturing total factor productivity developed steadily. From 2004 to 2019, the annual average of pharmaceutical manufacturing total factor productivity in China was 1.1422, that is, the average growth rate was 14.22%, and the cumulative HTFP was 4.1758. The trend of time change in the three regions is similar to that of the whole country, and the overall trend of time change in the three regions is small.

According to Fig. 5b, the spatial distribution of pharmaceutical manufacturing total factor productivity decreased from west to east, and from high to low, it was the west (1.1652), the middle (1.1357) and the east (1.1239). During the study period, the pharmaceutical industry in the western region has developed rapidly, while the eastern region has a strong level of development and is difficult to upgrade, so it is in a stable development trend. Within the region, in the eastern region, Beijing, Shanghai and Liaoning are at a higher level of development, but there is a big gap between their surrounding provinces and central cities; In the central region, the development of each province is relatively average, and the development difference between the surrounding provinces and the central cities is relatively small; In the western region, there is a big gap in the development of various provinces, Ningxia, Gansu and Yunnan are developing faster, while Shaanxi and Guangxi are developing slower.

To sum up, it is important to consider the aggregation of provinces and the heterogeneity of regions in the study of the effect of ER on pharmaceutical manufacturing total factor productivity.

**Figure 6:**
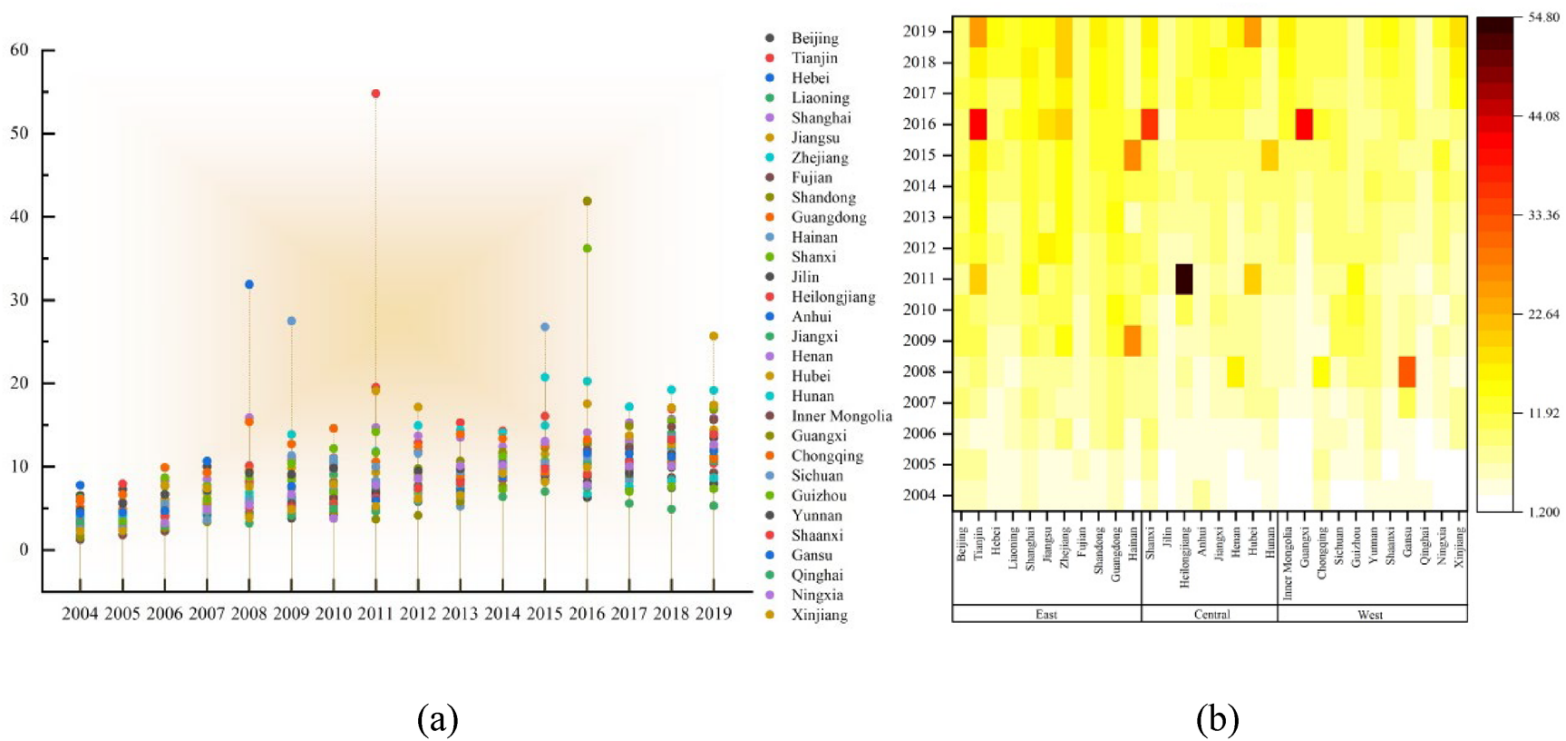
Temporal and spatial characteristics of ER.

Figure 6a and b describe the temporal and spatial variation characteristics of environmental regulation (ER). Figure 6a describes the change trend and aggregation characteristics of each province over time. Figure 6b describes trends in the spatial dynamics of ER by region and province from 2004 to 2019.

From Fig. 6a, the overall ER of the whole country shows a gradual upward trend over time, indicating that during the study period, the intensity of environmental regulation is increasing. Except for some provinces, most provinces show the spatial agglomeration characteristics of "from agglomeration to decentralization". Before 2014, the difference of ER in each province is small, but after 2014, the development of ER in each province is more dispersed. The differences in energy utilization, industrial pollution and resource allocation in each province continue to show, which leads to the different intensity of environmental regulation.

From Fig. 6b, the ER of each region and province shows a gradual upward trend over time, which is consistent with the overall change of the whole country. From the regional point of view, the average ER from 2004 to 2019 is the eastern (10.2144), the central (8.6867) and the western (7.8871), and the eastern ER is far more than that of the central and western regions, which shows that the eastern region has strengthened environmental regulation while developing its economy. This is closely related to the eastern industrial structure, pollution prevention and control, environmental input and so on. For example, in the eastern region, Tianjin (14.4987) and Zhejiang (12.5606) are in the leading position, while Fujian (7.1645), Liaoning (7.6985) and Hebei (7.8748) are relatively low; In the central region, Shanxi (11.5473) ranks first, while Jilin (5.5154) ER is not only at the lowest level in the central region, but also relatively backward in the whole country. There is a large range of "high-high" spatial aggregation of ER in the eastern and central regions, while the ER in the western region is generally low, which shows a "low-low" aggregation characteristics.

Therefore, in the follow-up study, we not only need to consider the local impact of ER, but also need to determine whether there is a spatial spillover effect, so as to more comprehensive analysis of the impact of ER on pharmaceutical manufacturing total factor productivity.

### Empirical results of panel data regression

Before studying the spatial spillover effects of ER on pharmaceutical manufacturing total factor productivity, we need to establish a panel data regression model. The effect of ER on pharmaceutical manufacturing total factor productivity was discussed. In this paper, ER is taken as the core explanatory variable and pharmaceutical manufacturing total factor productivity as the explained variable. By constructing mixed panel regression model (OLS), fixed effects model (FE) and random effects model (RE), the optimal regression model is selected. Table 3 is the regression results of the three models. Model-1, Model-2 and Model-3 are the regression results of mixed OLS, fixed effects and random effects, respectively. Model-4 is the result of fixed effect regression with ER squared. In Model-4, the regression coefficient of the square term of ER (ER^2) is not significant, so the impact of environmental regulation on the total factor productivity of pharmaceutical manufacturing industry is not a U-shaped relationship. Through the F test, LM test and Hausman test, the fixed effects model (Model-2) is the best model, and the goodness of fit of Model-2 is the best, which is 0.199, indicating that the explanatory degree of explanatory variables to HTFP is 19.9%.

**Table 3:** Results of baseline regression of HTFP by ER.

Model	(1) OLS	(2) FE	(3) RE	(4)FE
ER	12.31***	16.50***	16.85***	59.34***
	(3.795)	(3.288)	(3.250)	(7.632)
ER^2				− 1.103
				(0.179)
Open	0.438	1.186	0.252	0.984
	(0.640)	(0.948)	(0.777)	(0.912)
Labour	− 1.334	− 0.630	− 2.670	− 1.841
	(1.209)	(2.507)	(1.772)	(2.416)
Capital	0.795***	0.660***	0.638***	0.496***
	(0.169)	(0.165)	(0.159)	(0.161)
Income	− 0.913***	− 0.708***	− 0.705***	− 0.649***
	(0.205)	(0.186)	(0.184)	(0.179)
Profit	4.414**	4.083**	4.634***	4.137**
	(2.006)	(1.689)	(1.664)	(1.623)
Constant	293.3***	152.5**	240.6***	− 23.14
	(41.27)	(69.39)	(66.10)	(72.49)
F test		10.63***		11.67***
LM test			432.78***	508.13***
Hausman test		74.93***		3.78
R-squared	0.110	0.199	0.1932	0.262

**, ***indicate significance at the 5% and 1% level, and the standard errors are in parentheses.

From a fixed effect (Model-2), the regression coefficient of ER to pharmaceutical manufacturing total factor productivity was 16.5, which was significant at the level of 1, pharmaceutical manufacturing total factor productivity will rise by 16.5, which shows that the increase of environmental regulation has significantly promoted the improvement of total factor productivity in the pharmaceutical industry, proving that Hypothesis 1. On the one hand, according to the "compensation effect" and stricter environmental regulations, pharmaceutical enterprises that fail to meet the standards upgrade pollution prevention, they have to control technology, improve energy efficiency, reduce pollution emissions, which will improve the total factor productivity of pharmaceutical. On the other hand, according to the "reverse effect", pharmaceutical enterprises face the deepening environmental regulation and the increasing cost of pharmaceutical enterprise governance. It will make some enterprises continuously improve production efficiency under the pressure of environmental regulation, and upgrade production technology, industrial structure, resource energy consumption, environmental pollution and other aspects, so as to improve the total factor productivity of pharmaceutical.

From the impact of control variables on pharmaceutical manufacturing total factor productivity, Capital and Profit are significantly positive at the level of 1% and 5%, respectively. When Capital and Profit increase by 1, pharmaceutical manufacturing total factor productivity will increase by 0.660 and 4.083. The effect of Income on pharmaceutical manufacturing total factor productivity was significantly negative at 1% level. This shows that opening to the outside world and the profits of the whole industry are important factors to promote the development of pharmaceutical industry, while business income and the number of employees of industrial enterprises have less effect on improving pharmaceutical manufacturing total factor productivity.

### Empirical results of spatial econometric model

Through the analysis of temporal and spatial characteristics of ER and pharmaceutical manufacturing total factor productivity, this paper needs to further discuss whether there is spatial spillover effect of ER on pharmaceutical manufacturing total factor productivity. This part is divided into two parts. First, build the space weight matrix test and see whether there is spatial autocorrelation between ER and pharmaceutical manufacturing total factor productivity. Second, if there is spatial autocorrelation between ER and pharmaceutical manufacturing total factor productivity, the corresponding spatial econometric model is selected to study the spatial spillover effect of ER on pharmaceutical manufacturing total factor productivity.

#### Spatial autocorrelation test

Considering the accuracy and feasibility of the follow-up regression results, this paper constructs 0–1 adjacency matrix (W1) and geographical distance matrix (W2) as the basis of the follow-up study. In this paper, we test the spatial autocorrelation of pharmaceutical manufacturing total factor productivity and ER by global Moran index. The value of global Moran index is between − 1 and 1, and any value greater than 0 indicates the existence of positive spatial autocorrelation of the variable, and any value less than 0 indicates the existence of negative spatial autocorrelation of the variable. The global Moran exponents for W1 and W2 matrices are shown in Table 4. The results show that pharmaceutical manufacturing total factor productivity and ER have significant positive spatial autocorrelation.

**Table 4:** Global Moran index results of HTFP and ER.

Year	HTFP(W1)	HTFP(W2)	ER(W1)	ER(W2)
2004	0.237***	0.056***	0.298***	0.07***
2005	0.281***	0.051**	0.306***	0.066***
2006	0.325***	0.104***	0.286***	0.058***
2007	0.162**	− 0.002	0.269***	0.05**
2008	0.179**	0.043**	0.27***	0.046**
2009	0.201**	0.006	0.263***	0.044**
2010	0.242***	0.072***	0.254***	0.04**
2011	0.127*	0.013*	0.249***	0.039**
2012	0.256***	0.097***	0.258***	0.039**
2013	0.285***	0.112***	0.262***	0.039**
2014	0.236***	0.114***	0.267***	0.039**
2015	0.187**	0.095***	0.273***	0.039**
2016	0.223***	0.106***	0.277***	0.039**
2017	0.215**	0.089***	0.278***	0.037**
2018	0.207**	0.096***	0.279***	0.035**
2019	0.242***	0.084***	0.278***	0.034**

*, **, ***indicate significance at the 10%, 5% and 1% level, and the standard errors are in parentheses.

**Figure 7:**
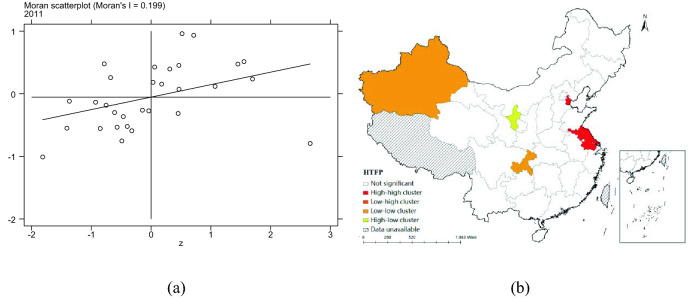
Moran scatter plot and LISA aggregation plot of HTFP.

**Figure 8:**
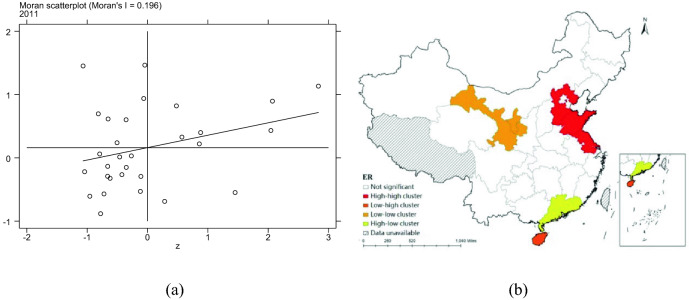
Moran scatter plot and LISA aggregation plot of ER.

In order to further analyze the spatial aggregation of each province, this paper calculates the local Moran index, draws the local Moran index scatter plot and the local autocorrelation LISA plot. The Moran scatter plot and LISA cluster plot of 2011 are drawn based on W1 matrix. Figure 7a and b are the Moran scatter plot and LISA of pharmaceutical manufacturing total factor productivity, respectively. Figure 8a and b are Moran scatter plot and LISA cluster plot of ER, respectively.

From the Moran scatter diagram of pharmaceutical manufacturing total factor productivity and ER, it can be seen that most provinces are concentrated in the first quadrant and the third quadrant, which indicates that there are obvious "high-high" or "low-low" aggregation in pharmaceutical manufacturing total factor productivity and ER. From the LISA cluster map of pharmaceutical manufacturing total factor productivity and ER, it can be seen that there is a "high-high" cluster of pharmaceutical manufacturing total factor productivity in Beijing and Jiangsu, a "low-low" cluster in Xinjiang and Chongqing, and a "high-low" cluster in Ningxia. ER in Hebei, Shandong and Jiangsu have "high-high" aggregation, Gansu and Ningxia have "low-low" aggregation, Guangdong has "high-low" aggregation and Hainan has "low–high" aggregation.

From Fig. 7a and b, the Moran scatter plot and LISA aggregation plot are shown that pharmaceutical manufacturing total factor productivity has the spatial agglomeration characteristics of "high-high" or "low-low" aggregation. For example, from Fig. 7a and b, pharmaceutical manufacturing total factor productivity shows "high-high" aggregation of provinces such as Tianjin and Jiangsu, and "low-low" aggregation of provinces such as Xinjiang and Chongqing. China's pharmaceutical manufacturing industry has a good momentum of development, under the influence of policies, resources and other factors, the overall showing a more obvious regional characteristics. In recent years, remarkable industrial clusters have been formed in the Yangtze River Delta, Dawan District and Bohai Rim, mainly relying on regional innovation-driven, industrial support, economic base and other advantages. The improvement of pharmaceutical manufacturing total factor productivity in the surrounding areas will promote the improvement of local pharmaceutical manufacturing total factor productivity to a certain extent Tianjin and Jiangsu, as representatives of the Bohai Rim and Yangtze River Delta, show the characteristics of "high-high" aggregation.

As an emerging industrial cluster, Sichuan-Chongqing region has a "low-low" aggregation in the results, the main reasons are: (1) there is a short-term effect of R&D investment on the growth of enterprises, but the R&D investment of enterprises needs long-term accumulation; (2) The innovation output cycle of pharmaceutical products is longer than that of other industries, and the innovation achievements may not be obvious in a short time; (3) There is innovation spillover effect in pharmaceutical manufacturing industry, and technology leaders provide technology to transferees involuntarily, which makes technology leaders fail to receive corresponding returns. Xinjiang borders Qinghai, Gansu and Inner Mongolia, and its geographical location is located in the westernmost part of China, far from the industrial cluster cities, forming a "low-low" agglomeration situation.

From Fig. 8a and b, the provinces with "high-high" ER are Hebei, Shandong and Jiangsu, and the provinces with "low-low" ER are Gansu and Ningxia. Hebei and Shandong are traditional provinces with large industrial and resource reserves, and also have severe environmental conditions, which require more stringent environmental regulation. So these industrial clusters have formed a "high- high" cluster.

In Jiangsu Province, which is close to the traditional industrial agglomeration area, the environmental regulation has also appeared the characteristics of "high-high" agglomeration. The main reasons are as follows: (1) Jiangsu is located in the border area of traditional industries, and there may be some enterprises using the layout of other places to avoid supervision, which aggravates the environmental pollution in the adjacent areas, so more stringent environmental regulation is needed; (2) Environmental regulation in Jiangsu has "marginal effect", and the environmental benefits brought by the same environmental input cost will be lower than other provinces, so more targeted and effective environmental governance measures should be taken to improve the efficiency of environmental regulation. However, Gansu and Ningxia are far away from traditional industrial clusters and heavy industrial clusters, and the intensity of environmental regulation is relatively small, thus forming the "low-low" aggregation characteristics of environmental regulation.

#### Regression results of spatial econometric model

In the above research, due to the existence of "compensation effect" and "inversion effect", increasing environmental regulation has a significant role in promoting the total factor productivity of the pharmaceutical industry. However, due to the significant spatial autocorrelation between ER and pharmaceutical manufacturing total factor productivity, there are obvious "high-high" or "low-low" aggregation characteristics in the region, so it is very important to study the spatial spillover of ER to pharmaceutical manufacturing total factor productivity. In the process of establishing the spatial econometric model, pharmaceutical manufacturing total factor productivity is taken as the explained variable, ER is taken as the core explanatory variable, and 0–1 adjacency matrix (W1) is taken as the spatial weight matrix to establish the spatial autoregressive model.

Table 5 is the regression results of three spatial econometric models. Model-5, Model-6 and Model-7 are the regression results of SAR, SEM and SDM, respectively. The results of LR test show that the spatial Durbin model (Model-7) is the optimal model. In addition, the goodness of fit of Model-7 was the best, which is 0.19, indicating that the explanatory degree of explanatory variables to HTFP was 19%.

**Table 5:** Result of spatial econometric model.

Model	(5) SAR	(6) SEM	(7) SDM
W*HTFP	0.344***	0.360***	0.187***
	(0.0528)	(0.0651)	(0.0595)
ER	9.751***	7.480**	6.481**
	(3.170)	(3.543)	(3.116)
Open	1.516*	1.981**	1.357
	(0.865)	(0.862)	(0.834)
Labour	− 1.213	− 2.435	− 2.044
	(2.286)	(2.413)	(2.269)
Capital	0.528***	0.482***	0.310**
	(0.152)	(0.163)	(0.156)
Income	− 0.715***	− 0.823***	− 0.644***
	(0.170)	(0.181)	(0.175)
Profit	4.647***	6.504***	5.087***
	(1.542)	(1.687)	(1.618)
W*ER			36.96***
			(5.736)
W*Open			− 2.728**
			(1.247)
W*Labour			2.429
			(2.818)
W*Capital			− 0.0265
			(0.209)
W*Income			0.591**
			(0.271)
W*Profit			− 6.195***
			(2.246)
sigma2_e	87,919***	90,097***	79,830***
	(5,737)	(5,923)	(5,171)
Log-likelihood	− 3420.292	− 3426.857	− 3392.086
LR test for SAR			56.41***
LR test for SEM			69.54***
Observations	480	480	480
R-squared	0.058	0.051	0.190
Number of ID	30	30	30

*, **, ***indicate significance at the 10%, 5% and 1% level, and the standard errors are in parentheses.

In this paper, the results of spatial Durbin model are used as the basis for the follow-up analysis.

First, the coefficient of pharmaceutical manufacturing total factor productivity spatial lag is 0.187, which is significant at 1% level. This shows that every 1 increase of HTFP in surrounding areas will increase HTFP by 0.187 in this area, which proves Hypothesis 2. The spatial lag term of pharmaceutical manufacturing total factor productivity is significantly positive, which also verifies the existence of spatial autocorrelation of pharmaceutical manufacturing total factor productivity, and the existence of "high-high" or "low-low" aggregation in each province. Under the national strategy of overall development of pharmaceutical industry, governments in various regions have intensified their policy efforts to support the development of pharmaceutical manufacturing industry in their respective regions from the perspectives of capital investment, talent attraction and infrastructure construction. Relying on the local resources to build the pharmaceutical manufacturing city, gradually deepen the degree of cluster industrialization, improve the competitiveness of pharmaceutical manufacturing enterprises in scale and innovation, so as to drive the development of pharmaceutical manufacturing total factor productivity in surrounding cities as a central city.

Second, the regression coefficient of ER's influence on local pharmaceutical manufacturing total factor productivity is 6.481, which is significant at 5%. This shows that environmental regulation has a significant role in promoting the improvement of local pharmaceutical manufacturing total factor productivity, which proves that Hypothesis 1. For every 1 increase in local ER, local pharmaceutical manufacturing total factor productivity will increase by 6.481. The regression coefficient of ER on local pharmaceutical manufacturing total factor productivity was 36.96, which was significant at 1%. This shows that the ER in the surrounding area has a significant role in improving the local pharmaceutical manufacturing total factor productivity, which proves that Hypothesis 2. For every 1 increase in ER in the surrounding area, the local pharmaceutical manufacturing total factor productivity will increase by 36.96. The role of environmental regulation on the total factor productivity of pharmaceutical manufacturing industry has a significant positive impact on both local and surrounding areas. On the one hand, according to the "backward effect", strengthening environmental regulation means that polluting pharmaceutical enterprises have high cost, forcing enterprises to carry out technological innovation, so as to improve the total productivity of pharmaceutical factors. On the other hand, environmental regulation will screen out "clean" pharmaceutical enterprises, so that the local formation of "green barriers". The "backward effect" makes the local green clean technology have a good demonstration effect on the surrounding areas, while the local "green barrier" transfers the non-clean enterprises to the surrounding areas to a certain extent, but because this transfer lags behind the "non-clean transfer" to pharmaceutical manufacturing total factor productivity.

Third, from the control variables on the impact of pharmaceutical manufacturing total factor productivity point of view, the control variable regression coefficient of the direction and fixed. Open, Capital and Profit have a positive effect on the local pharmaceutical manufacturing total factor productivity, but have a negative effect on the surrounding pharmaceutical manufacturing total factor productivity. Labor and Income have a negative impact on the local pharmaceutical manufacturing total factor productivity, but have a positive impact on the surrounding pharmaceutical manufacturing total factor productivity. The potential reason is that the central city absorbs the resource advantages of the surrounding areas, resulting in a "siphon effect", which leads to the lack of sufficient resources in the surrounding areas to improve the efficiency level of the pharmaceutical industry in the region.

### Further analysis

#### Mediating transmission mechanism

In this paper, the spatial spillover effect of ER on pharmaceutical manufacturing total factor productivity is studied by establishing a SDM. However, the impact of environmental regulation on the development of pharmaceutical manufacturing industry is not a direct relationship between the two, and there is a complex intermediary effect between them. Therefore, in the further analysis, this paper first studies the mediating effect between the two.

This paper divides the mediating effect path into technical effect and structural effect, and the technical effect is divided into green technology and production technology, so this paper selects Ingrva, RD and Structure as three mediating variables, which correspond to green technology, production technology and structural effect respectively. The results of the mediating effect under the spatial Durbin model are Tables 6, 7 and 8.

**Table 6:** Mediating effects of green technology.

Model	(8) Ingrva	(9) HTFP	(10) HTFP
W*HTFP	0.201***	0.274***	0.167***
	(0.0595)	(0.0562)	(0.0604)
ER	0.511**		5.826*
	(0.202)		(3.118)
Ingrva		0.203	− 0.00991
		(0.707)	(0.692)
Open	− 0.0252	1.300	1.321
	(0.0550)	(0.851)	(0.829)
Labour	0.218	− 2.715	− 2.725
	(0.151)	(2.335)	(2.273)
Capital	0.0210**	0.433***	0.276*
	(0.0104)	(0.158)	(0.156)
Income	− 0.0134	− 0.678***	− 0.596***
	(0.0116)	(0.179)	(0.175)
Profit	0.0428	5.203***	4.949***
	(0.107)	(1.652)	(1.610)
W*ER	0.729*		31.68***
	(0.373)		(6.044)
W*Ingrva		5.485***	3.170***
		(1.188)	(1.228)
W*Open	0.0443	− 3.603***	− 3.335***
	(0.0833)	(1.294)	(1.261)
W*Labour	0.324*	1.681	0.721
	(0.189)	(2.954)	(2.881)
W*Capital	0.0252*	− 0.0422	− 0.159
	(0.0140)	(0.219)	(0.214)
W*Income	− 0.0241	0.786***	0.753***
	(0.0182)	(0.284)	(0.276)
W*Profit	− 0.0437	− 7.016***	− 6.463***
	(0.149)	(2.292)	(2.235)
sigma2_e	349.9***	83,189***	78,831***
	(22.68)	(5408)	(5103)
R-squared	0.007	0.138	0.229

*, **, ***indicate significance at the 10%, 5% and 1% level, and the standard errors are in parentheses.

**Table 7:** Mediating effects of production technology.

Model	(11) RD	(12) HTFP	(13) HTFP
W*HTFP	0.275***	0.309***	0.190***
	(0.0554)	(0.0545)	(0.0595)
ER	2.448**		5.935*
	(1.121)		(3.089)
RD		0.304**	0.334***
		(0.127)	(0.124)
Open	− 2.018***	1.066	0.904
	(0.305)	(0.879)	(0.855)
Labour	− 5.162***	− 4.253*	− 4.591**
	(0.834)	(2.408)	(2.342)
Capital	0.357***	0.596***	0.415***
	(0.0572)	(0.161)	(0.160)
Income	0.445***	− 0.716***	− 0.549***
	(0.0641)	(0.183)	(0.180)
Profit	− 2.864***	4.691***	4.204***
	(0.590)	(1.671)	(1.626)
W*ER	3.738*		32.37***
	(2.026)		(5.849)
W*RD		1.077***	0.744***
		(0.201)	(0.203)
W*Open	− 1.766***	− 0.845	− 1.864
	(0.473)	(1.339)	(1.312)
W*Labour	3.594***	11.97***	7.577**
	(1.085)	(3.061)	(3.063)
W*Capital	− 0.297***	− 0.0208	− 0.219
	(0.0766)	(0.214)	(0.211)
W*Income	0.413***	− 0.0751	0.275
	(0.106)	(0.305)	(0.302)
W*Profit	− 1.253	− 4.086*	− 4.716**
	(0.839)	(2.344)	(2.282)
sigma2_e	10,718***	81,705***	77,132***
	(696.7)	(5321)	(4996)
R-squared	0.585	0.081	0.189

*, **, ***indicate significance at the 10%, 5% and 1% level, and the standard errors are in parentheses.

**Table 8:** Mediating effects of industrial structure.

Model	(14) Structure	(15) HTFP	(16) HTFP
W*HTFP	0.393***	0.266***	0.188***
	(0.0560)	(0.0549)	(0.0589)
ER	2.782		3.366
	(3.715)		(2.995)
Structure		0.178***	0.173***
		(0.0373)	(0.0368)
Open	− 4.512***	1.047	0.972
	(1.006)	(0.817)	(0.806)
Labour	0.243	− 1.064	− 1.570
	(2.745)	(2.181)	(2.153)
Capital	1.495***	0.409**	0.333**
	(0.192)	(0.161)	(0.160)
Income	0.539**	− 0.565***	− 0.498***
	(0.211)	(0.169)	(0.167)
Profit	− 11.89***	3.870**	3.542**
	(1.950)	(1.609)	(1.587)
W*ER	14.42**		24.08***
	(6.833)		(5.846)
W*Structure		0.583***	0.473***
		(0.0636)	(0.0678)
W*Open	− 0.513	− 1.170	− 1.623
	(1.518)	(1.210)	(1.197)
W*Labour	− 1.248	6.560**	4.385
	(3.422)	(2.682)	(2.692)
W*Capital	0.113	− 0.703***	− 0.681***
	(0.268)	(0.227)	(0.223)
W*Income	− 0.212	0.550**	0.603**
	(0.328)	(0.260)	(0.257)
W*Profit	1.473	− 2.797	− 3.236
	(2.744)	(2.201)	(2.174)
sigma2_e	116,880***	73,899***	71,762***
	(7674)	(4801)	(4648)
R-squared	0.482	0.171	0.252

*, **, ***indicate significance at the 10%, 5% and 1% level, and the standard errors are in parentheses.

Table 6 shows the regression day results with Ingrva as the mediator. ER is the core explanatory variables of Model-8, and the dependent variable is Ingrva. In Model-9, Ingrva was used as the core explanatory variable and pharmaceutical manufacturing total factor productivity was used as the dependent variable. ER and Ingrva were used as the core explanatory variables and pharmaceutical manufacturing total factor productivity as the dependent variable in Model-10.

According to Model-8, the spatial lag coefficient of Ingrva is 0.201, which is significant at 1% level. For every 1 increase in local Ingrva, the surrounding Ingrva will increase by 0.201. The regression coefficient of ER on local Ingrva was 0.511, which was significant at 5% level. The regression coefficient of the influence of ER in surrounding areas on local ln Ingrva is 0.729, significant at 10% level. This shows that increasing environmental regulation not only improves the local green technology level, but also improves the green technology level of the surrounding areas, and green technology has the effect of diffusion to the surrounding areas.

According to Model-9, the spatial lag coefficient of pharmaceutical manufacturing total factor productivity is 0.274, the regression coefficient of Ingrva to local pharmaceutical manufacturing total factor productivity is 0.203, and the regression coefficient of Ingrva to local pharmaceutical manufacturing total factor productivity in surrounding areas is 5.485. The effect of Ingrva on pharmaceutical manufacturing total factor productivity in the local area and the effect on the surrounding area both are positive, which indicates that Ingrva promotes the improvement of pharmaceutical manufacturing total factor productivity.

According to Model-10, the spatial lag coefficient of pharmaceutical manufacturing total factor productivity is 0.167, the regression coefficients of ER and Ingrva to local pharmaceutical manufacturing total factor productivity are 5.826 and − 0.00991, respectively, and the regression coefficients of ER and Ingrva to local pharmaceutical manufacturing total factor productivity are 31.68 and 3.17, respectively. Except the effect of Ingrva on local pharmaceutical manufacturing total factor productivity was not significant, the other response coefficients were significant. Both ER and Ingrva act on pharmaceutical manufacturing total factor productivity, that is, peripheral ER has a direct effect on local pharmaceutical manufacturing total factor productivity and an indirect effect through the action of Ingrva.

Through the above analysis, we can get the intermediate transmission path of Ingrva: the increase of environmental regulation in the region and surrounding areas promotes the improvement of local green technology, thus promoting the improvement of pharmaceutical manufacturing total factor productivity in the region and surrounding areas, which proves that Hypothesis 3.

Table 7 represents the regression result of RD as a mediation variable. Model-11 takes ER as the core explanatory variable and RD as the dependent variable. Model-12 takes RD as the core explanatory variable and pharmaceutical manufacturing total factor productivity as the dependent variable. Model-13 uses ER and RD as the core explanatory variables and pharmaceutical manufacturing total factor productivity as the dependent variable.

According to Model-11, the spatial lag coefficient of RD is 0.275, which is significant at the level of 1%. For every 1 increase in local RD, RD in surrounding areas will increase by 0.275. The regression coefficient of ER to local RD was 2.448, which was significant at 5%. The regression coefficient of ER to local RD in the surrounding area was 3.738, which was significant at 10%. This shows that increasing environmental regulation not only improves the local technological innovation, but also improves the technological innovation of the surrounding areas, and technological innovation has the effect of diffusion to the surrounding areas.

According to Model-12, the spatial lag coefficient of pharmaceutical manufacturing total factor productivity is 0.309, the regression coefficient of RD to local pharmaceutical manufacturing total factor productivity is 0.304, and the regression coefficient of RD to local pharmaceutical manufacturing total factor productivity in surrounding areas is 1.077, all of which are significant. RD has a negative impact on pharmaceutical manufacturing total factor productivity in the region and has a positive the surrounding areas.

According to Model-13, the spatial lag coefficient of pharmaceutical manufacturing total factor productivity is 0.190, the regression coefficients of ER and RD to local pharmaceutical manufacturing total factor productivity are 5.935 and 0.334 respectively, and the regression coefficients of ER and RD to local pharmaceutical manufacturing total factor productivity are 32.37 and 0.744 respectively in surrounding areas. All regression coefficients were significant. The peripheral ER not only had a direct impact on local pharmaceutical manufacturing total factor productivity, but also had an indirect impact through the role of RD.

Through the above analysis, we can get the intermediary transmission path of RD: the increase of environmental regulation in the region and surrounding areas promotes the improvement of local production technology, thus promoting the improvement of pharmaceutical manufacturing total factor productivity in the region and surrounding areas, which proves Hypothesis 3.

Table 8 shows the regression results with Structure as the mediating variable. In Model-14, ER is used as the core explanatory variable and Structure is used as the dependent variable. In Model-15, Structure was used as the core explanatory variable, and pharmaceutical manufacturing total factor productivity was used as the dependent variable. In Model-16, ER and Structure were used as core explanatory variables, and pharmaceutical manufacturing total factor productivity was used as dependent variable.

According to Model-14, the spatial lag coefficient of Structure is 0.393, which is significant at the 1% level. For every 1 increase in the local Structure, the surrounding area Structure will increase by 0.393. The regression coefficient of the effect of ER on local Structure is 2.782. The regression coefficient of the influence of ER in surrounding areas on local Structure is 14.42, which is significant at the level of 5%. This shows that the environmental regulation of surrounding areas has a certain degree of impact on the changes of pharmaceutical industrial structure, while the impact of local environmental regulation is not significant.

According to Model-15, the spatial lag coefficient of pharmaceutical manufacturing total factor productivity is 0.266, the regression coefficient of Structure to local pharmaceutical manufacturing total factor productivity is 0.178, and the regression coefficient of Structure to local pharmaceutical manufacturing total factor productivity is 0.583. This shows that pharmaceutical manufacturing total factor productivity in this area is not only affected by Structure in this area, but also affected the surrounding areas.

According to Model-16, the spatial lag coefficient of pharmaceutical manufacturing total factor productivity is 0.188, the regression coefficients of ER and Structure to local pharmaceutical manufacturing total factor productivity are 3.366 and 0.173, respectively. The regression coefficients of pharmaceutical manufacturing total factor productivity were 24.08 and 0.473 respectively. The regression coefficients of pharmaceutical manufacturing total factor productivity were significant except for the effect of ER on the local pharmaceutical manufacturing total factor productivity.

Through the above analysis, we can get the intermediary transmission path of Structure: the improvement of environmental regulation in surrounding areas promotes the upgrading of local pharmaceutical industrial structure, thus promoting the improvement of local pharmaceutical manufacturing total factor productivity, which proves that Hypothesis 3.

#### Heterogeneity analysis

Due to the differences of temporal and spatial variation characteristics of ER and pharmaceutical manufacturing total factor productivity in the eastern, central and western regions, the spatial spillover effects of ER on pharmaceutical manufacturing total factor productivity will also be different in different regions. Therefore, this paper takes into account the spatial agglomeration and geographic location heterogeneity of provinces in different regions, establishes a spatial Durbin model, and discusses the impact of ER on pharmaceutical manufacturing total factor productivity in the eastern, central and western regions.

Table 9 is the regression result of heterogeneity analysis. Model-7 is the regression result of the whole country, which is used as the control group of heterogeneity analysis. Model-17, Model-18 and Model-19 are the regression results of the eastern, central and western, respectively.

**Table 9:** Result of heterogeneous regression.

Model	(6) SDM	(16) East	(17) Central	(18) West
W*HTFP	0.187***	− 0.0136	0.546***	− 0.0228
	(0.0595)	(0.104)	(0.0523)	(0.0833)
ER	6.481**	7.755	0.342	5.550
	(3.116)	(8.325)	(1.433)	(3.888)
Open	1.357	0.825	− 5.734***	− 12.20***
	(0.834)	(1.193)	(1.965)	(3.452)
Labour	− 2.044	− 11.96***	16.75***	− 6.182
	(2.269)	(3.963)	(2.093)	(7.554)
Capital	0.310**	0.153	0.174	2.451***
	(0.156)	(0.230)	(0.236)	(0.690)
Income	− 0.644***	− 0.593**	0.846***	− 1.060*
	(0.175)	(0.255)	(0.239)	(0.621)
Profit	5.087***	7.733***	− 9.322***	6.178
	(1.618)	(2.531)	(1.727)	(4.454)
W*ER	36.96***	53.63***	2.615	1.434
	(5.736)	(13.08)	(1.666)	(6.579)
W*Open	− 2.728**	− 1.614	7.409***	12.61
	(1.247)	(1.614)	(2.345)	(8.141)
W*Labour	2.429	4.405	− 13.05***	23.48**
	(2.818)	(3.972)	(2.209)	(11.22)
W*Capital	− 0.0265	0.0962	0.236	− 0.137
	(0.209)	(0.278)	(0.261)	(1.691)
W*Income	0.591**	0.258	− 0.557*	1.006
	(0.271)	(0.346)	(0.310)	(1.286)
W*Profit	− 6.195***	− 5.647*	5.560***	− 4.949
	(2.246)	(2.938)	(1.958)	(7.502)
sigma2_e	79,830***	148,533***	5,155***	32,304***
	(5171)	(15,835)	(682.9)	(3444)
Observations	480	176	128	176
R-squared	0.190	0.123	0.657	0.330
Number of ID	30	11	8	11

*, **, ***indicate significance at the 10%, 5% and 1% level, and the standard errors are in parentheses.

According to the spatial lag coefficient of pharmaceutical manufacturing total factor productivity, the influence degree of pharmaceutical manufacturing total factor productivity in the surrounding areas on local pharmaceutical manufacturing total factor productivity is only significant in the central.

According to the regression coefficient of the impact of local ER on local pharmaceutical manufacturing total factor productivity, three regions are all not significant. From the regression coefficient of the impact of ER on local pharmaceutical manufacturing total factor productivity in the surrounding areas, only the impact of eastern (53.63) is positive and significant at the level of 1%.

To sum up, the spatial spillover effect of the three regions is not obvious by analyzing the heterogeneous characteristics of the three regions alone. This further verifies the necessity of taking 30 provinces in China as a whole as the research object.

#### Endogenous discussion

Although the use of spatial econometric model can better study the spatial spillover effect of ER and pharmaceutical manufacturing total factor productivity, there may be endogenous problems caused by the omission of variables and the results of bias, so this paper uses GS2SLS spatial econometric tool variable method to alleviate the endogenous problems that may exist in the model.

Based on Hering and Poncet (2014), this paper uses *Ventilation* as the instrumental variable of environmental regulation^79^. According to Jacobson (2003), the air flow coefficient is equal to the product of the boundary layer height and the wind speed^80^. In this paper, based on the global network of ten meters wind speed and boundary layer height data in the ERA-Interim database of the European Center for Medium-Range Weather Forecasts, the air circulation coefficient of each network in the corresponding year is calculated, and then the air circulation coefficient of each province is obtained according to the longitude and latitude matching of each provincial capital city.

When air pollutant emissions are the same, cities with low air ventilation coefficient tend to use more stringent environmental regulation tools. The calculation process of environmental regulation itself includes environmental pollution, so it can be considered that there is a correlation between environmental regulation and air circulation coefficient. Moreover, the air circulation coefficient only depends on natural phenomena such as climate conditions, and there is no other mechanism with the total factor productivity of the pharmaceutical industry, so the air circulation coefficient as an instrumental variable, which has exogeneity.

Appendix Table 5 is the result of the GS2SLS instrumental variable method. From the results, Ventilation coefficient and environmental regulation (ER) are significantly negative at the level of 10%, with a coefficient of − 3.165. The results are in agreement with the theoretical expectation.

The spatial lag coefficient of ER is 0.0842, but the result is not significant, which indicates that local environmental regulation is endogenous, while the environmental regulation of surrounding areas is not endogenous, indicating that there is no two-way causal relationship between ER of surrounding areas and local pharmaceutical manufacturing total factor productivity.

#### Robustness test

In order to test the robustness of the model established in this paper, the following methods are used to compare the robustness of the results by replacing the control variables and the spatial weight matrix. Appendix Table 6 shows the results of the robustness test. Model-7 was the control group, Model-21 replaced the control variable Open with Open2, Model-22 replaced the control variable Capital with Capital2, and Model-23 replaced the 0–1 adjacency matrix (W1) with the geographical distance matrix (W2). As a result, the magnitude and significance of the regression coefficients changed only slightly, but not in direction. Therefore, the spatial econometric regression results obtained in this paper are robust.

In addition, the results reported in this article are the absolute form of all variables. In order to better verify the robustness of the model, the results of all logarithmic and mixed variables are reported in the appendix (Appendix Tables 1–5). These results have great consistency in model selection and the direction of main variables, but there are some differences in the significance of variables.

## Conclusions and policy recommendations

In this paper, the direct effect and spatial spillover effect of ER on pharmaceutical manufacturing total factor productivity are studied by establishing fixed effect model and spatial Dubin model. The main conclusions are as follows:

First, increasing environmental regulation has a significant role in promoting the total factor productivity of pharmaceutical manufacturing industry before ER reaches the limit. For every 1% increase in ER, pharmaceutical manufacturing total factor productivity will increase by 0.751%.

Second, ER and pharmaceutical manufacturing total factor productivity have significant positive spatial autocorrelation, and there are "high-high" aggregation and "low-low" aggregation characteristics. Environmental regulation has a significant positive impact on the total factor productivity of pharmaceutical manufacturing industry in local and surrounding areas before ER reaches the limit. For every 1% increase in ER, the local pharmaceutical manufacturing total factor productivity will increase by 0.101%, and the surrounding pharmaceutical manufacturing total factor productivity will increase by 0.553%.

Third, in the further analysis, environmental regulation has an impact on pharmaceutical manufacturing total factor productivity through the intermediary effects of green technology, production technology and industrial structure. At the same time, the effect of ER on pharmaceutical manufacturing total factor productivity shows heterogeneous characteristics in the eastern, central and western regions.

Based on the above empirical results, this paper puts forward the following policy recommendations from the perspective of the whole country and the three major regions:

Firstly, for the whole country, facing the urgent requirements of clean industry transformation, it is necessary to combine the development characteristics and advantages of each region, strengthen regional cooperation and promote collaborative governance. Accelerate the construction of integrated demonstration zones, radiate the development of surrounding areas, and promote the coordinated development of pharmaceutical manufacturing industry. At the same time, it is necessary to build a scientific research platform to improve the ability of independent innovation. Encourage and guide enterprises, colleges and universities and other innovative subjects to make full use of their scientific research advantages. Actively build a platform for cultivating and sharing scientific research achievements, optimize the scientific research and innovation environment by means of strategic cooperation, and expand the transformation and application of scientific and technological achievements, so as to improve green technology and production technology.

Secondly, for the eastern region, we need to effectively use the regional economic environment, infrastructure, technological innovation, industrial clusters and other advantages to optimize the industrial structure of pharmaceutical manufacturing industry and develop environmental protection industry. With the concept of green development, we should promote green pharmaceutical manufacturing industry, develop green circular economy, and constantly improve the scientific and technological content and clean content of the industry. At the same time, we need to standardize the market order and improve the trading mechanism. Drawing on mature experience, we should actively cultivate a platform for property rights trading of environmental resources, promote the implementation of market-oriented environmental and economic policy mechanisms such as green insurance and green credit, and promote the level of total factor productivity of pharmaceutical manufacturing industry with a mild environmental regulation system.

Thirdly, for the central region, we should focus on technological transformation and technological innovation, vigorously promote the transformation and upgrading of traditional industries, accelerate the transformation of green and clean industries and the development of pharmaceutical manufacturing industries, improve the productivity of all manufacturing workers, upgrade the level of industrial development, and promote the transformation of central speed to central quality. At the same time, we should improve the industrial complementarity of the central region, expand the small regional urban agglomeration to large-scale industrial clusters, and give full play to the advantages of each region, so as to improve the promoting effect of environmental regulation on improving the total factor productivity of pharmaceutical manufacturing industry.

Finally, for the western region, the role of environmental regulation in promoting the development of pharmaceutical manufacturing industry is limited, the backward effect to improve the technological innovation of enterprises is insufficient in terms of funds, and the environmental regulation of surrounding areas will make the region a pollution-bearing area, which requires the government to increase financial support for clean transformation enterprises, such as reducing taxes and costs. Implement restrictive policies on enterprises, increasing the cost of cross-regional transfer of polluting enterprises, limiting the number of cross-regional transfer of polluting enterprises, and increasing government investment in R&D, fundamentally solving the problem of transformation of pollution enterprises, so as to improve the total factor productivity of pharmaceutical manufacturing industry.

## Supplementary Information

Supplementary Information.

## Data Availability

The data selected in this paper are all from China Statistical Yearbook, China Environmental Yearbook, China High-tech Industry Statistical Yearbook, China Environmental Statistical Yearbook, China Industrial Statistical Yearbooks, China Health Statistical Yearbooks, provincial statistical yearbooks and the ERA-Interim database of the European Center for Medium-Range Weather Forecasts. The data that support the findings of this study are available from [www.cnki.net], but restrictions apply to the availability of these data, which were used under license for the current study, and so are not publicly available. Data are however available from the authors upon reasonable request and with permission of [www.cnki.net].
