# Supplementary material for: The spatial spillover effect of environmental regulation on the total factor productivity of pharmaceutical manufacturing industry in China
Source: Sci Rep. 2022 Jul 8;12:11642. doi: 10.1038/s41598-022-15614-8 (PMC9264754; doi:10.1038/s41598-022-15614-8)
Supplement: Supplementary file 1 — Supplementary Information. [file 41598_2022_15614_MOESM1_ESM.docx]

**Appendix A**

**Appendix Table 1. Result of the reference regression of the logarithmic form and the mixed form of variables**

| Model | (1) OLS | (2) FE | (3) RE | (4)FE |
| --- | --- | --- | --- | --- |
|  | Logarithmic form | | | |
| lnER | 0.527*** | 0.225*** | 0.360*** | 0.316 |
|  | (0.0652) | (0.0535) | (0.0545) | (0.195) |
| lnER^2 |  |  |  | -0.0206 |
|  |  |  |  | (0.0425) |
| lnOpen | -0.300*** | -0.0447 | -0.131*** | -0.0434 |
|  | (0.0366) | (0.0346) | (0.0354) | (0.0347) |
| lnLabour | -0.0655 | -0.415*** | -0.469*** | -0.419*** |
|  | (0.0431) | (0.112) | (0.0792) | (0.112) |
| lnCapital | 0.482*** | 0.720*** | 0.694*** | 0.716*** |
|  | (0.133) | (0.114) | (0.118) | (0.114) |
| lnIncome | -0.0655 | -0.0284 | -0.0833 | -0.0322 |
|  | (0.147) | (0.125) | (0.130) | (0.126) |
| lnProfit | 0.0871 | 0.132*** | 0.131** | 0.131** |
|  | (0.0734) | (0.0510) | (0.0549) | (0.0511) |
| Constant | 2.994*** | 2.555*** | 3.018*** | 2.512*** |
|  | (0.271) | (0.317) | (0.272) | (0.330) |
| F test |  | 28.89*** |  | 27.28*** |
| LM test |  |  | 685.01*** | 666.35*** |
| Hausman test |  | 138.60*** |  | 119.04*** |
| Observations | 480 | 480 | 480 | 480 |
| R-squared | 0.372 | 0.701 | 0.6843 | 0.701 |
|  | Mixed form | | | |
| lnER | 0.686*** | 0.751*** | 0.791*** | 1.941*** |
|  | (0.0618) | (0.0491) | (0.0493) | (0.191) |
| lnER^2 |  |  |  | -0.296*** |
|  |  |  |  | (0.0460) |
| Open | -0.000542 | 0.00232* | -0.000481 | 0.00241* |
|  | (0.00101) | (0.00131) | (0.00114) | (0.00125) |
| Labor | -0.00276 | 0.00494 | -0.00377 | 0.00195 |
|  | (0.00190) | (0.00349) | (0.00265) | (0.00337) |
| Capital | 0.000770*** | 0.000827*** | 0.000643*** | 0.000856*** |
|  | (0.000266) | (0.000230) | (0.000229) | (0.000220) |
| Income | -0.000983*** | -0.000752*** | -0.000658** | -0.000815*** |
|  | (0.000323) | (0.000258) | (0.000262) | (0.000247) |
| Profit | 0.00697** | 0.00481** | 0.00679*** | 0.00561** |
|  | (0.00315) | (0.00233) | (0.00237) | (0.00224) |
| Constant | 4.329*** | 3.748*** | 4.007*** | 2.702*** |
|  | (0.128) | (0.118) | (0.132) | (0.198) |
| F test |  | 18.26*** |  | 19.12*** |
| LM test |  |  | 700.11*** | 739.18*** |
| Hausman test |  | 21.22*** |  | 18.57*** |
| Observations | 480 | 480 | 480 | 480 |
| R-squared | 0.285 | 0.552 | 0.535 | 0.590 |

**Appendix Table 2. Spatial results of logarithmic and mixed forms of variables**

| Model | (5) SAR | (6) SEM | (7) SDM |
| --- | --- | --- | --- |
|  | Logarithmic form | | |
| W*HTFP | 0.492*** | 0.317*** | 0.319*** |
|  | (0.0457) | (0.0905) | (0.0551) |
| lnER | 0.0967** | 0.173*** | 0.0509 |
|  | (0.0469) | (0.0529) | (0.0465) |
| lnOpen | -0.0243 | -0.0591* | -0.00187 |
|  | (0.0294) | (0.0344) | (0.0313) |
| lnLabour | -0.322*** | -0.401*** | -0.295*** |
|  | (0.0950) | (0.108) | (0.102) |
| lnCapital | 0.367*** | 0.703*** | 0.0802 |
|  | (0.102) | (0.112) | (0.112) |
| lnIncome | -0.0705 | -0.0562 | -0.108 |
|  | (0.106) | (0.118) | (0.104) |
| lnProfit | 0.135*** | 0.115** | 0.120*** |
|  | (0.0432) | (0.0486) | (0.0434) |
| W*lnER |  |  | 0.277*** |
|  |  |  | (0.0902) |
| W*lnOpen |  |  | 0.0236 |
|  |  |  | (0.0554) |
| W*lnLabour |  |  | 0.102 |
|  |  |  | (0.147) |
| W*lnCapital |  |  | 0.222 |
|  |  |  | (0.179) |
| W*lnIncome |  |  | 0.130 |
|  |  |  | (0.174) |
| W*lnProfit |  |  | -0.0139 |
|  |  |  | (0.0688) |
| Log-likelihood | -135.8281 | -173.7774 | -106.0845 |
| LR test for SAR |  |  | 59.49*** |
| LR test for SEM |  |  | 135.39*** |
| sigma2_e | 0.0967*** | 0.118*** | 0.0889*** |
|  | (0.00638) | (0.00781) | (0.00579) |
| R-squared | 0.200 | 0.122 | 0.161 |
|  | Mixed form | | |
| W*HTFP | 0.674*** | 0.820*** | 0.460*** |
|  | (0.0320) | (0.0216) | (0.0455) |
| lnER | 0.196*** | -0.00539 | 0.101** |
|  | (0.0428) | (0.0441) | (0.0430) |
| Open | 0.00329*** | 0.00461*** | 0.00286*** |
|  | (0.000901) | (0.000779) | (0.000861) |
| Labor | 0.00247 | -0.000917 | 0.00271 |
|  | (0.00240) | (0.00223) | (0.00235) |
| Capital | 0.000511*** | 0.000383** | 0.000379** |
|  | (0.000159) | (0.000152) | (0.000160) |
| Income | -0.000831*** | -0.000964*** | -0.000792*** |
|  | (0.000177) | (0.000178) | (0.000180) |
| Profit | 0.00526*** | 0.00692*** | 0.00537*** |
|  | (0.00160) | (0.00165) | (0.00166) |
| W*ER |  |  | 0.553*** |
|  |  |  | (0.0662) |
| W*Open |  |  | -0.00648*** |
|  |  |  | (0.00128) |
| W*Labor |  |  | 0.000975 |
|  |  |  | (0.00295) |
| W*Capital |  |  | -0.000380* |
|  |  |  | (0.000213) |
| W*Income |  |  | 0.00115*** |
|  |  |  | (0.000278) |
| W*Profit |  |  | -0.00597*** |
|  |  |  | (0.00232) |
| sigma2_e | 0.0954*** | 0.0925*** | 0.0843*** |
|  | (0.00635) | (0.00621) | (0.00554) |
| Log-likelihood | -149.6041 | -165.076 | -100.635 |
| LR test for SAR |  |  | 97.94*** |
| LR test for SEM |  |  | 128.88*** |
| R-squared | 0.125 | 0.010 | 0.393 |

**Appendix Table 3. The mediating effect of variable logarithmic form and mixed form**

| Model | (8)  lnIngrva | (9)  lnHTFP | (10)  lnHTFP | (11)  lnRD | (12)  lnHTFP | (13)  lnHTFP | (14)  lnStructure | (15)  lnHTFP | (16)  lnHTFP |
| --- | --- | --- | --- | --- | --- | --- | --- | --- | --- |
|  | Logarithmic form | | | | | | | | |
| W*Y | 0.0911 | 0.334*** | 0.309*** | 0.280*** | 0.264*** | 0.248*** | 0.610*** | 0.125** | 0.120* |
|  | (0.0637) | (0.0551) | (0.0560) | (0.0633) | (0.0594) | (0.0598) | (0.0376) | (0.0636) | (0.0637) |
| lnER | 0.0107 |  | 0.0525 | 0.166** |  | 0.0337 | -0.0222 |  | 0.0273 |
|  | (0.0302) |  | (0.0463) | (0.0783) |  | (0.0462) | (0.0321) |  | (0.0447) |
| lnM |  | -0.136* | -0.135* |  | 0.0820*** | 0.0775*** |  | 0.325*** | 0.324*** |
|  |  | (0.0700) | (0.0694) |  | (0.0275) | (0.0273) |  | (0.0648) | (0.0647) |
| lnOpen | -0.0809*** | -0.0140 | -0.0121 | 0.0555 | -0.0113 | -0.00933 | 0.0468** | -0.0166 | -0.0155 |
|  | (0.0205) | (0.0320) | (0.0317) | (0.0532) | (0.0314) | (0.0311) | (0.0216) | (0.0300) | (0.0299) |
| lnLabour | 0.0164 | -0.365*** | -0.294*** | -0.699*** | -0.287*** | -0.224** | 0.517*** | -0.398*** | -0.358*** |
|  | (0.0670) | (0.0997) | (0.102) | (0.174) | (0.101) | (0.103) | (0.0714) | (0.104) | (0.106) |
| lnCapital | 0.314*** | 0.181 | 0.126 | 0.212 | 0.138 | 0.0886 | 0.198** | -0.0138 | -0.0441 |
|  | (0.0735) | (0.114) | (0.114) | (0.190) | (0.112) | (0.112) | (0.0777) | (0.107) | (0.108) |
| lnIncome | -0.115* | -0.149 | -0.129 | 0.280 | -0.203* | -0.180* | -0.0745 | -0.134 | -0.118 |
|  | (0.0682) | (0.105) | (0.104) | (0.178) | (0.106) | (0.106) | (0.0717) | (0.0992) | (0.0990) |
| lnProfit | 0.0237 | 0.142*** | 0.123*** | -0.157** | 0.153*** | 0.136*** | -0.0439 | 0.156*** | 0.144*** |
|  | (0.0284) | (0.0432) | (0.0432) | (0.0736) | (0.0431) | (0.0432) | (0.0299) | (0.0410) | (0.0413) |
| W*lnER | -0.00956 |  | 0.284*** | 0.206 |  | 0.261*** | 0.0893 |  | 0.189** |
|  | (0.0589) |  | (0.0901) | (0.152) |  | (0.0894) | (0.0624) |  | (0.0864) |
| W*lnM |  | -0.0962 | -0.133 |  | 0.114* | 0.108* |  | 0.238*** | 0.210** |
|  |  | (0.135) | (0.135) |  | (0.0599) | (0.0595) |  | (0.0883) | (0.0894) |
| W*lnOpen | -0.0347 | 0.0107 | 0.000561 | -0.167* | 0.0607 | 0.0529 | -0.0727* | 0.00316 | 0.00154 |
|  | (0.0370) | (0.0580) | (0.0577) | (0.0942) | (0.0559) | (0.0555) | (0.0381) | (0.0534) | (0.0533) |
| W*lnLabour | -0.0477 | 0.121 | 0.110 | 0.322 | 0.107 | 0.0906 | -0.696*** | 0.393*** | 0.376*** |
|  | (0.0963) | (0.148) | (0.147) | (0.249) | (0.147) | (0.146) | (0.101) | (0.146) | (0.146) |
| W*lnCapital | 0.00705 | 0.511*** | 0.281 | -0.603** | 0.464*** | 0.248 | 0.134 | 0.0644 | -0.0690 |
|  | (0.118) | (0.174) | (0.186) | (0.300) | (0.165) | (0.179) | (0.126) | (0.167) | (0.176) |
| W*lnIncome | 0.0374 | -0.0232 | 0.113 | 1.285*** | -0.201 | -0.0641 | 0.385*** | -0.137 | -0.0436 |
|  | (0.114) | (0.171) | (0.174) | (0.298) | (0.175) | (0.180) | (0.120) | (0.162) | (0.166) |
| W*lnProfit | -0.0185 | 0.0265 | -0.0118 | -0.177 | 0.0678 | 0.0299 | -0.113** | 0.190*** | 0.156** |
|  | (0.0449) | (0.0683) | (0.0686) | (0.117) | (0.0688) | (0.0694) | (0.0483) | (0.0682) | (0.0695) |
| sigma2_e | 0.0382*** | 0.0897*** | 0.0881*** | 0.255*** | 0.0886*** | 0.0872*** | 0.0422*** | 0.0807*** | 0.0798*** |
|  | (0.00247) | (0.00586) | (0.00574) | (0.0166) | (0.00576) | (0.00566) | (0.00280) | (0.00521) | (0.00516) |
| R-squared | 0.087 | 0.082 | 0.119 | 0.302 | 0.145 | 0.200 | 0.764 | 0.198 | 0.235 |
|  | Mixed form | | | | | | | | |
| W*Y | 0.128** | 0.705*** | 0.464*** | 0.368*** | 0.425*** | 0.319*** | 0.724*** | 0.215*** | 0.183*** |
|  | (0.0621) | (0.0305) | (0.0454) | (0.0527) | (0.0516) | (0.0561) | (0.0303) | (0.0599) | (0.0608) |
| lnER | 0.0448 |  | 0.104** | 0.155** |  | 0.0602 | 0.0823** |  | 0.0171 |
|  | (0.0285) |  | (0.0431) | (0.0711) |  | (0.0430) | (0.0330) |  | (0.0426) |
| lnM |  | -0.0409 | -0.0870 |  | 0.135*** | 0.119*** |  | 0.236*** | 0.231*** |
|  |  | (0.0681) | (0.0657) |  | (0.0279) | (0.0272) |  | (0.0601) | (0.0594) |
| Open | -0.000264 | 0.00329*** | 0.00284*** | -0.00176 | 0.00302*** | 0.00275*** | -0.00152** | 0.00387*** | 0.00352*** |
|  | (0.000593) | (0.000890) | (0.000859) | (0.00143) | (0.000870) | (0.000846) | (0.000657) | (0.000830) | (0.000826) |
| Labor | 0.000983 | 0.00254 | 0.00279 | 0.00705* | 0.000159 | 0.000995 | 0.0133*** | -0.000450 | -0.000239 |
|  | (0.00163) | (0.00244) | (0.00235) | (0.00395) | (0.00238) | (0.00232) | (0.00179) | (0.00238) | (0.00236) |
| Capital | 5.60e-05 | 0.000601*** | 0.000385** | 0.000141 | 0.000490*** | 0.000344** | -1.23e-05 | 0.000229 | 0.000199 |
|  | (0.000111) | (0.000165) | (0.000160) | (0.000268) | (0.000160) | (0.000157) | (0.000123) | (0.000157) | (0.000155) |
| Income | -0.000110 | -0.000930*** | -0.000804*** | 0.000321 | -0.00103*** | -0.000861*** | 0.000518*** | -0.000983*** | -0.000907*** |
|  | (0.000125) | (0.000187) | (0.000181) | (0.000301) | (0.000180) | (0.000176) | (0.000137) | (0.000174) | (0.000173) |
| Profit | 0.000735 | 0.00551*** | 0.00545*** | -0.00600** | 0.00740*** | 0.00656*** | -0.00655*** | 0.00840*** | 0.00785*** |
|  | (0.00115) | (0.00172) | (0.00166) | (0.00279) | (0.00168) | (0.00164) | (0.00127) | (0.00164) | (0.00163) |
| W*lnER | 0.0924** |  | 0.559*** | 0.570*** |  | 0.423*** | 0.285*** |  | 0.268*** |
|  | (0.0379) |  | (0.0688) | (0.104) |  | (0.0701) | (0.0525) |  | (0.0736) |
| W*lnM |  | 0.372*** | 0.00492 |  | 0.259*** | 0.139*** |  | 0.358*** | 0.250*** |
|  |  | (0.119) | (0.121) |  | (0.0469) | (0.0498) |  | (0.0726) | (0.0785) |
| W*Open | 0.00129 | -0.00627*** | -0.00636*** | 0.00199 | -0.00568*** | -0.00634*** | -0.00218** | -0.00419*** | -0.00473*** |
|  | (0.000894) | (0.00134) | (0.00129) | (0.00216) | (0.00130) | (0.00126) | (0.000989) | (0.00125) | (0.00124) |
| W*Labor | 0.00140 | 0.00496 | 0.00111 | 0.00322 | 0.00239 | -2.03e-05 | -0.00937*** | 0.00398 | 0.00262 |
|  | (0.00205) | (0.00304) | (0.00295) | (0.00495) | (0.00295) | (0.00289) | (0.00226) | (0.00288) | (0.00287) |
| W*Capital | 0.000469*** | -0.000188 | -0.000338 | -8.08e-06 | -0.000114 | -0.000309 | 0.000252 | -0.000591*** | -0.000613*** |
|  | (0.000149) | (0.000224) | (0.000216) | (0.000357) | (0.000213) | (0.000209) | (0.000163) | (0.000207) | (0.000205) |
| W*Income | -0.000503*** | 0.00111*** | 0.00110*** | 0.000940** | 0.000514* | 0.000807*** | 7.51e-05 | 0.000712*** | 0.000847*** |
|  | (0.000194) | (0.000294) | (0.000283) | (0.000473) | (0.000285) | (0.000280) | (0.000214) | (0.000269) | (0.000269) |
| W*Profit | 0.00158 | -0.00720*** | -0.00582** | -0.00347 | -0.00318 | -0.00392* | 0.000379 | -0.00101 | -0.00187 |
|  | (0.00161) | (0.00240) | (0.00232) | (0.00392) | (0.00238) | (0.00231) | (0.00180) | (0.00230) | (0.00230) |
| sigma2_e | 0.0407*** | 0.0908*** | 0.0839*** | 0.237*** | 0.0859*** | 0.0808*** | 0.0494*** | 0.0786*** | 0.0767*** |
|  | (0.00263) | (0.00606) | (0.00551) | (0.0155) | (0.00564) | (0.00527) | (0.00331) | (0.00510) | (0.00497) |
| R-squared | 0.015 | 0.118 | 0.346 | 0.241 | 0.297 | 0.394 | 0.607 | 0.318 | 0.366 |

**Appendix Table 4. Heterogeneity analysis results of variable logarithmic form and mixed form**

| Model | (17) East | (18) Central | (19) West |
| --- | --- | --- | --- |
|  | Logarithmic form | | |
| W*lnHTFP | 0.0950 | 0.326*** | 0.240*** |
|  | (0.0858) | (0.0648) | (0.0917) |
| lnER | -0.138 | 0.0880* | 0.171** |
|  | (0.0889) | (0.0459) | (0.0750) |
| lnOpen | 0.117 | -0.147*** | -0.0663* |
|  | (0.112) | (0.0540) | (0.0393) |
| lnLabour | -0.572*** | 0.188 | -0.270 |
|  | (0.168) | (0.133) | (0.186) |
| lnCapital | 0.502** | 0.0607 | 0.0428 |
|  | (0.235) | (0.137) | (0.159) |
| lnIncome | -0.889*** | 0.484*** | 0.0617 |
|  | (0.249) | (0.143) | (0.145) |
| lnProfit | 0.545*** | -0.0880 | 0.0410 |
|  | (0.105) | (0.0559) | (0.0594) |
| W*lnER | 0.358** | -0.102* | -0.303** |
|  | (0.144) | (0.0580) | (0.133) |
| W*lnOpen | -0.179 | -0.0361 | 0.0358 |
|  | (0.184) | (0.0720) | (0.0681) |
| W*lnLabour | 0.00785 | -0.759*** | 0.478* |
|  | (0.195) | (0.136) | (0.264) |
| W*lnCapital | 0.602* | 0.514*** | 0.00502 |
|  | (0.348) | (0.165) | (0.282) |
| W*lnIncome | 0.502 | 0.0884 | 0.488* |
|  | (0.403) | (0.176) | (0.279) |
| W*lnProfit | -0.352*** | -0.0732 | 0.0311 |
|  | (0.134) | (0.0645) | (0.0983) |
| sigma2_e | 0.0748*** | 0.0233*** | 0.0969*** |
|  | (0.00799) | (0.00298) | (0.0104) |
| Observations | 176 | 128 | 176 |
| R-squared | 0.154 | 0.820 | 0.285 |
| Number of ID | 11 | 8 | 11 |
|  | Mixed form | | |
| W*lnHTFP | 0.336*** | 0.609*** | 0.241*** |
|  | (0.0754) | (0.0464) | (0.0778) |
| lnER | 0.00630 | 0.181*** | 0.248*** |
|  | (0.0872) | (0.0504) | (0.0697) |
| Open | 0.00228** | -0.0133*** | -0.0101* |
|  | (0.000908) | (0.00432) | (0.00564) |
| Labour | -0.00788*** | 0.0217*** | 0.00117 |
|  | (0.00295) | (0.00465) | (0.0125) |
| Capital | 0.000245 | -4.15e-05 | 0.00425*** |
|  | (0.000173) | (0.000525) | (0.00117) |
| Income | -0.000679*** | 0.00126** | -0.00268*** |
|  | (0.000189) | (0.000531) | (0.00103) |
| Profit | 0.00752*** | -0.0109*** | 0.0116 |
|  | (0.00187) | (0.00378) | (0.00716) |
| W*lnER | 0.899*** | 0.119* | -0.0350 |
|  | (0.116) | (0.0631) | (0.118) |
| W*Open | -0.00422*** | 0.0176*** | 0.0165 |
|  | (0.00121) | (0.00519) | (0.0133) |
| W*Labour | 0.00235 | -0.0155*** | 0.0605*** |
|  | (0.00302) | (0.00483) | (0.0190) |
| W*Capital | -3.04e-05 | 5.75e-06 | -0.00347 |
|  | (0.000207) | (0.000599) | (0.00304) |
| W*Income | 0.000576** | -0.000643 | 0.00497** |
|  | (0.000257) | (0.000679) | (0.00225) |
| W*Profit | -0.00616*** | 0.00855** | -0.0117 |
|  | (0.00220) | (0.00413) | (0.0121) |
| sigma2_e | 0.0821*** | 0.0254*** | 0.0837*** |
|  | (0.00900) | (0.00340) | (0.00897) |
| Observations | 176 | 128 | 176 |
| R-squared | 0.545 | 0.709 | 0.460 |
| Number of ID | 11 | 8 | 11 |

**Appendix Table 5. The endogeneity of the absolute form, the logarithmic form and the mixed form of variables**

| (20)Model | Absolute | Logarithmic | Mixed |
| --- | --- | --- | --- |
| Ventilation | -3.165* | -0.0819*** | -0.256** |
|  | (1.679) | (0.0313) | (0.109) |
| Open | 0.505 | -0.0234 | 2.92E-08 |
|  | (0.402) | (0.0343) | (0.0000000339) |
| Labor | -0.0154 | 0.0438 | 0.00104*** |
|  | (0.0234) | (0.0267) | (0.000315) |
| Capital | -0.0267 | -0.0257 | 9.78E-05 |
|  | (1.10) | (0.0771) | (0.0000915) |
| Income | 0.985 | 0.192** | 2.51E-05 |
|  | (1.61) | (0.0853) | (0.000137) |
| Profit | -0.637 | -0.174*** | -0.00354*** |
|  | (1.21) | (0.0409) | (0.00126) |
| W*ER | 0.0842 | 0.000735 | 0.0106 |
|  | (0.0543) | (0.00861) | (0.0172) |
| W*e.ER | -2.802*** | -2.654*** | -1.946** |
|  | (0.503) | (0.286) | (0.841) |
| Constant | 126.8*** | 4.518*** | 5.167*** |
|  | (8.618) | (0.180) | (0.18) |
| Pseudo R2 | 0.0775 | 0.1857 | 0.2838 |
| Wald test of spatial terms | 33.02*** | 89.99*** | 5.78* |
| Observations | 30 | 30 | 30 |

**Appendix Table 6. Robustness test results in the absolute form of the variables**

| Model | (7)Control  group | (21)ER-Investment | (22)Open-Open2 | (23)Capital-Capital2 | (24)W1-W2 |
| --- | --- | --- | --- | --- | --- |
| W*HTFP | 0.187*** | 0.319*** | 0.175*** | 0.0461 | -0.0393 |
|  | (0.0595) | (0.0534) | (0.0597) | (0.0645) | (0.145) |
| ER | 6.481** | -0.000451*** | 5.964* | 3.063 | 0.364 |
|  | (3.116) | (0.000135) | (3.138) | (3.081) | (3.164) |
| Open | 1.357 | 1.472* | -2.09e-06 | 1.873** | 1.028 |
|  | (0.834) | (0.843) | (4.79e-06) | (0.828) | (0.807) |
| Labour | -2.044 | -0.402 | -2.140 | -2.608 | -1.404 |
|  | (2.269) | (2.287) | (2.352) | (2.128) | (2.265) |
| Capital | 0.310** | 0.545*** | 0.281* | 0.00113 | 0.206 |
|  | (0.156) | (0.154) | (0.170) | (0.00147) | (0.152) |
| Income | -0.644*** | -0.826*** | -0.483*** | -0.527*** | -0.570*** |
|  | (0.175) | (0.174) | (0.164) | (0.142) | (0.172) |
| Profit | 5.087*** | 5.177*** | 4.846*** | 6.107*** | 4.305*** |
|  | (1.618) | (1.634) | (1.634) | (1.568) | (1.559) |
| W*ER | 36.96*** | 0.00108*** | 35.47*** | 19.80*** | 27.22** |
|  | (5.736) | (0.000180) | (5.791) | (6.169) | (12.45) |
| W*Open | -2.728** | -1.222 | -1.40e-05* | 0.151 | -3.193 |
|  | (1.247) | (1.296) | (7.29e-06) | (1.202) | (3.234) |
| W*Labour | 2.429 | 5.690** | 0.782 | 4.930* | -12.63* |
|  | (2.818) | (2.795) | (2.951) | (2.708) | (7.298) |
| W*Capital | -0.0265 | 0.211 | 0.356* | 0.00785*** | 0.556 |
|  | (0.209) | (0.206) | (0.212) | (0.00178) | (0.456) |
| W*Income | 0.591** | 0.202 | 0.383* | 0.0618 | 0.665 |
|  | (0.271) | (0.282) | (0.221) | (0.208) | (0.426) |
| W*Profit | -6.195*** | -3.903* | -6.411*** | -4.605** | -3.898 |
|  | (2.246) | (2.308) | (2.300) | (2.170) | (4.235) |
| sigma2_e | 79,830*** | 80,280*** | 80,480*** | 74,945*** | 75,646*** |
|  | (5,171) | (5,230) | (5,210) | (4,839) | (4,883) |
| R-squared | 0.190 | 0.043 | 0.185 | 0.185 | 0.245 |
